# Strontium and Copper Co-Doped Multifunctional Calcium Phosphates: Biomimetic and Antibacterial Materials for Bone Implants

**DOI:** 10.3390/biomimetics9040252

**Published:** 2024-04-20

**Authors:** Vladimir N. Lebedev, Mariya I. Kharovskaya, Bogdan I. Lazoryak, Anastasiya O. Solovieva, Inna V. Fadeeva, Abdulkarim A. Amirov, Maksim A. Koliushenkov, Farid F. Orudzhev, Oksana V. Baryshnikova, Viktoriya G. Yankova, Julietta V. Rau, Dina V. Deyneko

**Affiliations:** 1 Chemistry Department, Lomonosov Moscow State University, Leninskie Gory 1, 119991 Moscow, Russia; vladimir.lebedev@chemistry.msu.ru (V.N.L.); masha.harovskaaya@gmail.com (M.I.K.); bilazoryak@gmail.com (B.I.L.); sheoksana@yandex.ru (O.V.B.); 2 Laboratory of Pharmacology Active Compounds, Research Institute of Clinical and Experimental Lymphology–Branch of the Institute of Cytology and Genetics, Siberian Branch of Russian Academy of Sciences (RICEL–Branch of IC&G SB RAS), 630060 Novosibirsk, Russia; solovevaao@gmail.com; 3 A.A. Baikov Institute of Metallurgy and Material Science RAS, Leninskie, 49, 119334 Moscow, Russia; fadeeva_inna@mail.ru; 4 Amirkhanov Institute of Physics, Dagestan Scientific Center of Russian Academy of Sciences, 367003 Makhachkala, Russia; amiroff_a@mail.ru; 5 Physics Department, Lomonosov Moscow State University, Leninskie Gori 1, 119991 Moscow, Russia; koliushenkov.ma19@physics.msu.ru; 6 Geothermal and Renewal Energy Institute of the High Temperature Joint Institute of the Russian Academy of Sciences, 367015 Makhachkala, Russia; farid-stkha@mail.ru; 7 Institute of Pharmacy, Department of Analytical, Physical and Colloid Chemistry, I.M. Sechenov First Moscow State Medical University, Trubetskaya 8, Building 2, 119048 Moscow, Russia; yankova_v_g@staff.sechenov.ru (V.G.Y.); giulietta.rau@ism.cnr.it (J.V.R.); 8 Istituto di Struttura della Materia, Consiglio Nazionale delle Ricerche, ISM-CNR, Via del Fosso del Cavaliere 100, 00133 Rome, Italy; 9 Laboratory of Arctic Mineralogy and Material Sciences, Kola Science Centre RAS, 14 Fersman Str., 184209 Apatity, Russia

**Keywords:** calcium phosphate, TCP, whitlockite, antibacterial, biomimetic properties, bone substitutes

## Abstract

β-tricalcium phosphate (β-TCP) is a promising material in regenerative traumatology for the creation of bone implants. Previously, it was established that doping the structure with certain cations can reduce the growth of bacterial activity. Recently, much attention has been paid to co-doped β-TCP, that is explained by their ability, on the one hand, to reduce cytotoxicity for cells of the human organism, on the other hand, to achieve a successful antibacterial effect. Sr, Cu-co-doped solid solutions of the composition Ca_9.5–*x*_Sr*_x_*Cu(PO_4_)_7_ was obtained by the method of solid-phase reactions. The Rietveld method of structural refinement revealed the presence of Sr^2+^ ions in four crystal sites: M1, M2, M3, and M4. The M5 site is completely occupied by Cu^2+^. Isomorphic substitution of Ca^2+^ → (Sr^2+^and Cu^2+^) expands the concentration limits of the existence of the solid solution with the β-TCP structure. No additional phases were formed up to *x* = 4.5 in Ca_9.5–*x*_Sr*_x_*Cu(PO_4_)_7_. Biocompatibility tests were performed on cell lines of human bone marrow mesenchymal stromal cells (hMSC), human fibroblasts (MRC-5) and osteoblasts (U-2OS). It was demonstrated that cytotoxicity exhibited a concentration dependence, along with an increase in osteogenesis and cell proliferation. Ca_9.5–*x*_Sr*_x_*Cu(PO_4_)_7_ powders showed significant inhibitory activity against pathogenic strains Escherichia coli and Staphylococcus aureus. Piezoelectric properties of Ca_9.5–*x*_Sr*_x_*Cu(PO_4_)_7_ were investigated. Possible ways to achieve high piezoelectric response are discussed. The combination of bioactive properties of Ca_9.5–*x*_Sr*_x_*Cu(PO_4_)_7_ renders them multifunctional materials suitable for bone substitutes.

## 1. Introduction

The regenerative approach to restoring damaged bone tissues is emerging as a compelling and promising method in osteosurgery. This method involves utilizing implants with compositions similar to human bones. Calcium phosphate (CaP)-based substances have demonstrated efficacy as bone substitutes owing to their excellent biocompatibility. However, postoperative complications, often caused by harmful bacteria, remain a challenging issue to address.

To mitigate the proliferation of undesirable pathogens, compounds based on the β-tricalcium phosphate (β-Ca_3_(PO_4_)_2_, β-TCP) (space group *R*3*c*, unit cell parameters: *a* = *b* = 10.439 Å and *c* = 37.375 Å) [1] with various types of dopants applied to biomedical implants can prove useful. The low-temperature modification β-TCP has high biocompatibility [2], resorbable properties [3,4] and osteoinductive characteristics [5]. The crystallochemical aspect of β-TCP is also of considerable interest. The β-TCP structure consists of five cationic sites (M1–M5) [6,7]. The variety of crystallographic positions and rigid structure of phosphates allows for isomorphic substitutions of host Ca^2+^ ions by iso- or heterovalent ions, and co-doping, as well [8,9,10].

Copper ions (Cu^2+^) may become suitable candidates for partial replacement of calcium. Their action contributes not only to osteogenesis but also to a decrease in bacterial growth. Strong antibacterial properties of copper ions could prevent peri-implantitis—inflammation around a newly installed implant [11]. Previous studies have demonstrated a clear antibacterial effect, with a death rate of about 22% at a concentration of Cu^2+^ ions at approximately 12 mol.% in the β-TCP structure, and acceptable cytocompatibility [12]. However, such a bacteria death rate might not be sufficient, raising the question of how to enhance the antibacterial effectiveness.

One of the ways for increasing antibacterial activity while maintaining biocompatibility is through co-substitution in CPs [13,14,15]. Co-substituted CPs demonstrate a higher antibacterial effect than mono-substituted ones, without a negative effect on cell cultures [16], Ag and Zn or Ti, for instance. As a co-dopant, Strontium (Sr) is of interest because it can also inhibit several strains of microorganisms, such as *Escherichia coli* and *Staphylococcus aureus* [17,18]. Thereby, the antibacterial effect can be enhanced. Additionally, the presence of Sr^2+^ ions in the CPs-based implants can positively impact bone tissue due to their physiological similarity to calcium ions [19]. The presence of Sr^2+^ ions in the implant can stimulate osteogenesis [20], and increase adhesion, proliferation and differentiation of osteoblasts [21,22]. The controlled release of a specific amount of Sr^2+^ ions can facilitate the healing of osteoporotic bone defects [23]. Furthermore, it was shown that Sr-doped β-TCP scaffolds promote early angiogenesis [24]. Sr^2+^ ions prevented the formation of biofilms [25] without exhibiting cytotoxicity for mesenchymal stem cells [26].

Incorporation of Sr^2+^ ions into the β-TCP structure above 2.25 mol% also enhances the phase purity of the reaction product. This is achieved as the synthesis temperature range expands to 1125 °C, thereby preventing the α→β transition in the β-TCP structure [27]. The doping of the β-TCP structure by 10% mol. of Sr^2+^ ions does not affect the α-TCP phase stabilization [28].

In this work, to validate the hypothesis regarding the positive synergetic effect of Cu^2+^ and Sr^2+^ co-doping in the β-TCP, we synthesized a series of solid solutions Ca_9.5–*x*_Sr*_x_*Cu(PO_4_)_7_. Based on our previous findings [12], the concentration of Cu^2+^ ions was held constant at 9.5 mol.% to achieve a pronounced antibacterial effect. Meanwhile, the concentration of Sr^2+^ ions was varied in a wide range from 0 to 42 mol.% corresponding to the formula Ca_9.5–*x*_Sr*_x_*Cu(PO_4_)_7_. The area of isomorphic substitution was investigated and the crystal structures were refined using the Rietveld method. The bioactive properties, including cytotoxicity study and cell proliferation, of the Ca_9.5–*x*_Sr*_x_*Cu(PO_4_)_7_ powders were evaluated across various cell lines, including mesenchymal stromal cells (*h*MSC), human fibroblasts (MRC-5) and osteoblasts (U-2 OS). Additionally, the antibacterial effect was determined against *Escherichia coli* and *Staphylococcus aureus*. Biomimetic properties were also examined through the piezoelectric effect on the Ca_9.5–*x*_Sr*_x_*Cu(PO_4_)_7_ powders. Finally, the influence of the crystal structure and concentration of doping ions are discussed.

## 2. Materials and Methods

### 2.1. Synthetic Route

Strontium-copper-substituted phosphates with a general formula Ca_9.5–*x*_Sr*_x_*Cu(PO_4_)_7_ were obtained by solid-phase synthesis at high temperatures (Table 1). Before the synthesis, we calculated and weighed the initial quantities of substances according to the stoichiometry of the reaction. The initial reagents for solid-state synthesis CaHPO_4_⋅2H_2_O (99.9%), SrCO_3_ (99.9%), CaCO_3_ (99.9%), (NH_4_)_2_H_2_PO_4_ (99.9%) and CuO (99.9%) were purchased from the Sigma-Aldrich (Gillingram, UK). Raw reagents were mixed and homogenized. The mixtures were preheated at 773 K for 12 h with a low heating rate for slow removal of gas products. Then the samples were annealed at 1173 K and kept for 18 h. After that, the final stage of synthesis was carried out at 1173 K and kept for 18 h. Between each stage, grinding and homogenization was carried out at room temperature in the presence of acetone in an agate mortar [12]. Before grinding, the mixtures were slowly cooled. Synthesis of Sr^2+^, Cu^2+^-doped TCP was performed according to the following reactions:

14CaHPO_4_·2H_2_O + 2*x*SrCO_3_ + (5 − 2*x*)CaCO_3_ + 2CuO → 2Ca_9.5–*x*_Sr*_x_*Cu(PO_4_)_7_ + 5CO_2_↑ + 35H_2_O (0 ≤ *x* ≤ 4.5).

The parameters of the unit cells were calculated by describing the profile using the least squares method. The error is calculated using the charge flipping method (Table 1).

### 2.2. Characterization

#### 2.2.1. Powder X-Ray Diffraction Study

Powder X-ray diffraction (PXRD) patterns were collected on a Rigaku SmartLab SE: 3 kW sealed X-ray tube, D/teX Ultra 250 silicon strip detector (Rigaku, Tokyo, Japan), vertical type θ-θ geometry, CuKα radiation, HyPix-400 (2D HPAD) detector (Rigaku, Tokyo, Japan). PXRD data were collected at room temperature in the 2θ range between 3° and 80° with a step interval of 0.02°. The X-ray data were reproduced three times. The LeBail decomposition and structure refinement by the Rietveld methods were applied using the JANA2006 software (Version 2020) [29]. The crystal structures of Ca_9.5–*x*_Sr*_x_*Cu(PO_4_)_7_ samples were refined using the Rietveld method [30]. The crystal structure of Ca_9.5_Cu(PO_4_)_7_ (CSD Deposition Number 2220569) [12] served as a starting model for the refinement. The space group *R*3*c* was chosen [12,31]. The background was described by a Chebyshev polynomial function (31st order). Pseudo-Voigt functions were employed for fitting the reflection profiles. The refined structural parameters included the atomic coordinates and the isotropic temperature factor. The isotropic temperature factors were constrained to be the same for all oxygen atoms. The main crystallographic data from the Rietveld refinement and experimental details for Ca_9.5–*x*_Sr*_x_*Cu(PO_4_)_7_ samples are listed in Tables S3–S12 of the Supplementary Information.

#### 2.2.2. Fourier-Transform Infrared (FT-IR) Study

The FT-IR spectra of the samples were recorded on an FT-803 Fourier spectrometer (Simeks Research and Production Company 2022 Novosibirsk, Russia) in the wavenumber region of 4000–400 cm^−1^, with 1 cm^−1^ spectral resolution. The standard KBr disc method was applied to obtained the spectra.

#### 2.2.3. The Ion Release Behavior

For the study of Ca^2+^, Sr^2+^ and Cu^2+^ release into solutions behavior, the powder samples were pressed into pallets (d = 0.4 cm, m ~ 0.2 g). A hydraulic oil press was used to produce the tablets. The release of Ca^2+^, Sr^2+^ and Cu^2+^ from Ca_9.5–*x*_Sr*_x_*Cu(PO_4_)_7_ was investigated by soaking of Ca_9.5_Cu(PO_4_)_7_ and Ca_5_Sr_4.5_Cu(PO_4_)_7_ pallets in the 0.05 M Tris-HCl (pH = 7.4) buffer solution (75 mL). The soaking systems were placed in flasks for 4, 7, 14 and 18 days. The accumulative release amount of Ca^2+^, Sr^2+^ and Cu^2+^ ions was measured using inductively coupled plasma optical emission spectroscopy (ICP-OES, 720-ES axial spectrometer (Agilent Technologies, New York, NY, USA). The obtained data were reproduced three times and reported as mean ± standard deviation.

#### 2.2.4. In Vitro Biological Response to the Ceramics

Cytocompatibility of the studied samples was evaluated on human mesenchymal bone marrow stromal cells (MSCs), human lung fibroblasts (MRC-5) and U-2 OS (human osteosarcoma cell line, resemble pre-osteoblasts). Cells were cultured in Dulbecco’s modified Eagle’s medium (DMEM, Sigma Aldrich, St. Louis, MO, USA) supplemented with 10% fetal bovine serum (Gibco) under standard culture conditions (humidified atmosphere, 5% CO_2_ and 95% air, at 37 °C). Before the biocompatibility study, U-2 OS cell lines were differentiated into osteoblasts for a 3-week culture period. Differentiation was performed by adding ascorbic acid (50 μg/mL) on day 4 and β-glycerolphosphate (5 mM) on day 11 to the culture medium.

##### Cell Cultivation on a Powder Layer

**Figure 1:**
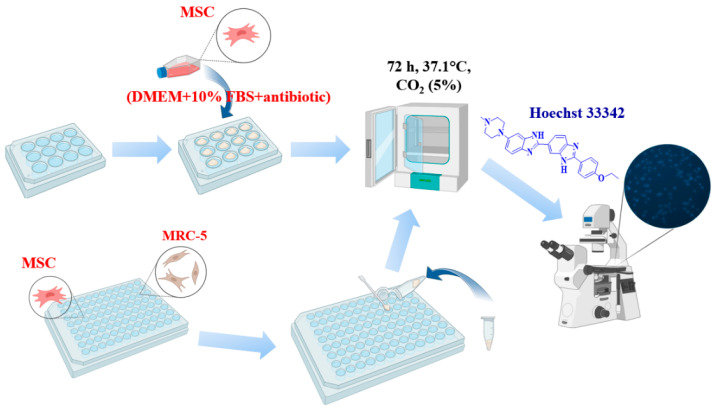
The sequence of research on biological cell cultures. Created with BioRender.com.

Sterile cover glasses with a diameter of 1 cm were placed in a 12-well plate, onto which powder samples were evenly applied in a thin continuous layer. The samples were then incubated with 50 µL of culture medium to obtain a dense layer. MSCs and MRC-5 cells were seeded at a density of 10 × 10^3^ per 100 mL/glass in medium (DMEM + 10%FBS + antibiotic/antimycotic). After 24 h, 1 mL of cellular medium was added, and the cells were incubated for 3 days. Analysis of cell number and viability was determined using fluorescent dyes Hoechst 33342 (a fluorescent DNA intercalating dye that stains the nuclei of all cells) and PI (stains only the nuclei of dead cells). Cells were analyzed by fluorescence microscopy (Zeiss, Axio observer Z1, Oberkochen, Germany). The acquired images were processed using ZEN blue software (ZEN3.1. blue edition) (Zeiss, Oberkochen, Germany) (Figure 1).

##### Adding Powders of Ceramics to Cell Medium

MSCs and MRC-5 cells were seeded in 96-well plates at a density of 1 × 10^4^ cells per 100 µL/well. After 24 h, powders were added on top of the cells into the wells, and the cells were then incubated with the powders for 3 days. Cell viability was assessed by fluorescent microscopy, with cell nuclei stained by Hoechst 33342.

The addition of elements such as Ca, Zn, Sr and Cu to bone implants is intended to improve their osteogenic properties. Copper is an essential metal in the human body including playing an important functional role in bone growth and development. It was shown that Cu at certain concentrations increases osteogenic differentiation of mesenchymal stromal cells (MSC) [32,33]. In turn, strontium is also an extremely important element in osteogenesis. Strontium ions (Sr^2+^) promote osteogenesis by enhancing the expression of osteogenesis-related genes in mesenchymal stem cells through activation of signaling pathways such as Wnt/β-catenin and RAS/MAPK [34]. And of course, calcium ions play an essential role in the formation of bone tissue. Ca^2+^ activates various intracellular signaling pathways such as yes-associated protein (YAP), wingless integration site (WNT) and mitogen-activated protein kinase (MAPK)—extracellular signal-regulated kinase (ERK)1/2. Through these pathways, Ca^2+^ stimulates the expression of osteogenic genes, osteoblast proliferation and MSCs differentiation [35,36].

So, the effect of ions released into the culture medium on cell viability was also determined. MSCs and U-2 OS cells were seeded in 96-well plates (1 × 10^4^ cells/100 µL/well). The powders (100 mg) were placed in 1.5 mL Eppendorf tubes, mixed with 1 mL of cell medium, and then incubated at 37 °C for 24 h. After incubation, the tubes containing the powder suspension were centrifuged at 10,000 g, and the supernatant containing diluted Cu^2+^, Sr^2+^ and Ca^2+^ cations was added to cells, replacing the old medium. Different concentrations were used, with a two-fold dilution step. The cells were incubated then for 2 days, after which the MTT test was performed. The MTT test based on the reduction of MTT reagent (3-(4,5-dimethylthiazol-2-yl)-2,5-diphenyltetrazolium) in the cell cytoplasm to a strongly light-absorbing formazan is among the most commonly used methods for the determination of cell viability and activity of NAD-dependent oxidoreductases. MTT test was performed according to the standard protocol. Optical density was measured using a Multiscan Sky device (Thermo Fisher Scientific Inc. Waltham, MA, USA) at a wavelength of 540 nm.

#### 2.2.5. Antimicrobial Activity Study

The antimicrobial activity of Ca_9.5–*x*_Sr*_x_*Cu(PO_4_)_7_ was investigated using *E. coli* and *S. aureus*. The study employed a dense nutrient-rich medium, GRM-agar, produced by the FSB SSC PMB (Obolensk, Russia). A physiological solution (PS), consisting of sodium chloride (NaCl = 9 g/l), was used as a buffer. Night cultures of the reference strains were fully looped and suspended in 0.5 mL of PS. Subsequently, 0.1 mL of all dilutions were seeded onto GRM-agar in Petri dishes to count the number of colony-forming units (CFU) at the beginning of the experiment (0 h). Each sample, weighing 0.1 g, was placed in a 1.5 mL Eppendorf test tube (Eppendorf, Germany) and 0.99 mL of PS was added. Additionally, PS (0.9 mL) was added to another test tube as a control, without the powder under investigation. The test tubes were sealed with Parafilm M sealing film (Bemis Company, Inc., USA) without closing the lid and shaken on a vortex for 2 min. The tubes were then incubated at 37 °C for 24 h. At the end of the incubation period, 0.1 mL of suspension was extracted from each tube, and 0.1 mL of all dilutions was plated onto GRM-agar in Petri dishes to count the number of CFUs (24 h).

#### 2.2.6. Piezoelectric Properties

The local piezoelectric properties were examined using the Piezoresponse Force Microscopy (PFM) technique with a commercial Ntegra II scanning probe microscope from NT-MDT Spectrum Instruments, Russia. Commercial cantilevers NSG01/Pt were used for these measurements. The HD-PFM scanning regime was employed to mitigate the undesirable effect of particle shifting by the tip. The modulation amplitude and frequency were set at 10 V and 550 kHz, respectively.

## 3. Results

### 3.1. PXRD Study

**Figure 2:**
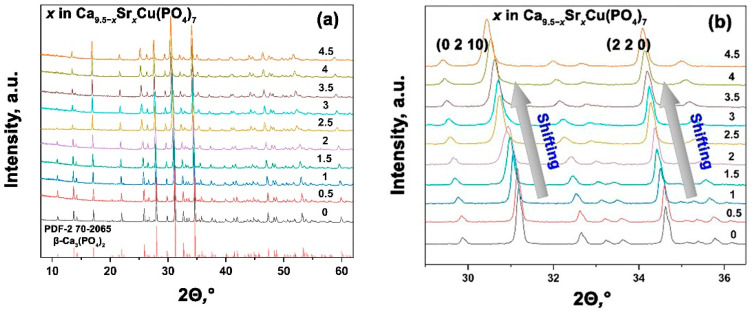
PXRD patterns of Ca_9.5–*x*_Sr*_x_*Cu(PO_4_)_7_ with 0 ≤ *x* ≤ 4.5 (**a**). Shifting of the main diffraction reflections in Ca_9.5–*x*_Sr*_x_*Cu(PO_4_)_7_, 0 ≤ *x* ≤ 4.5 (**b**).

The PXRD patterns of synthesized Ca_9.5–*x*_Sr*_x_*Cu(PO_4_)_7_ 0 ≤ *x* ≤ 4.5 solid solutions are presented in Figure 2a. The data analysis revealed that all the powder samples belong to the β-TPC structural type. No impurities from either the apatite type or pyrophosphate phases were detected, which confirms a complete reaction. In [37], the maximum doping concentrations of both Cu^2+^ and Sr^2+^ ions were ~ 2.52 mol.%. The absence of impurity phases confirmed the complete incorporation of these ions into the β-TCP structure, represented by the formula Ca_9.97_Sr_0.265_Cu_0.265_(PO_4_)_7_, as determinate by elemental analysis [37]. In the single Sr^2+^-doped solid solution Ca_10.5–*x*_Sr*_x_*(PO_4_)_7_ [31], the limit of Sr^2+^ substitution before a structural change was determinate to be *x* = 6, corresponding to Ca_4.5_Sr_6_(PO_4_)_7_. According to our results, the reduction of the unit cell caused by Cu^2+^ incorporation does not hinder the formation of single-phase solid solutions throughout the studied range in Ca_9.5–*x*_Sr*_x_*Cu(PO_4_)_7_ (0 ≤ *x* ≤ 4.5).

In the smaller-angle scale of the PXRD pattern, one can notice a shift in reflexes towards decreasing angles *θ*, that indicates an increase in the parameters of the unit cell. This effect correlates well with the classical Bragg law and confirms the successful isomorphic substitution in the structure (Figure 2b).

**Figure 3:**
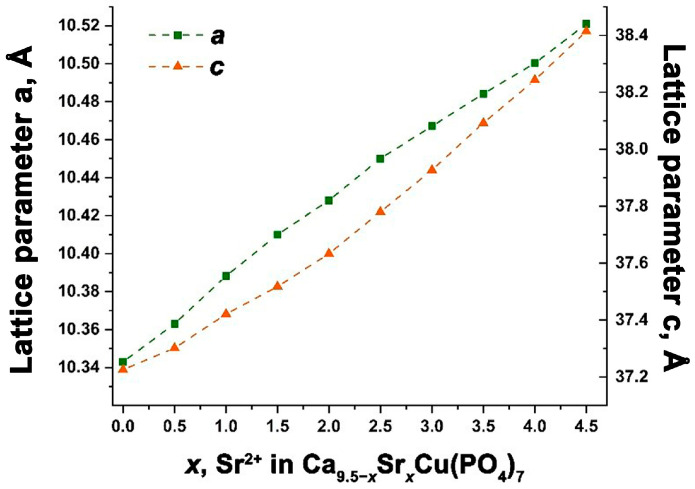
The dependence of the unit cell parameters *a* and *c* for Ca_9.5–*x*_Sr*_x_*Cu(PO_4_)_7_ solid solutions.

The calculation of the unit cells parameters *a*, *c* and the volumes *V* is consistent with the Vegard’s law. This suggests the creation of a continuous series of solid solutions Ca_9.5–*x*_Sr*_x_*Cu(PO_4_)_7_, confirming the intended isomorphic substitution (Figure 3). The precise values of the elementary cell parameters are detailed in Table 1.

### 3.2. Fourier-Transform Infrared Study

**Figure 4:**
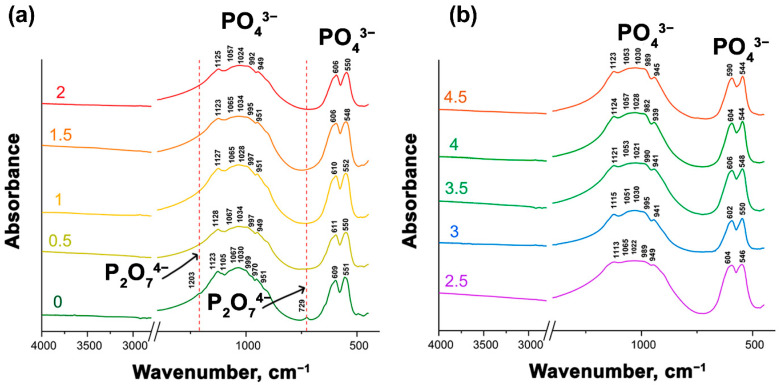
FT-IR spectra of Ca_9.5–*x*_Sr*_x_*Cu(PO_4_)_7_ with 0 ≤ *x* ≤ 2 (**a**) and 2 ≤ *x* ≤ 4.5 (**b**).

Figure 4a,b shows the FT-IR spectra of Ca_9.5–*x*_Sr*_x_*Cu(PO_4_)_7_. All the presented spectra are very similar. Absorption bands of PO_4_^3–^ groups are noticeable in all spectra. The vibrational modes in the phosphate stretching and bending regions appear in the range of 939–999 cm^−1^ for ν_1s_, 1128–1021 cm^−1^ for ν_3as_ and 611–544 cm^−1^ for *ν*_4_ [38]. However, transmission peaks at 1203 and 729 cm^−1^ were identified in Ca_9.5_Cu(PO_4_)_7_. These low-intensity bands suggest the presence of the ν [P–O–P] from P_2_O_7_^4–^. However, the PXRD patterns did not show reflections attributed to the pyrophosphate phase, its amount being below the sensitivity level of the instrument. Thus, P_2_O_7_^4–^ ions likely do not significantly contribute to the properties.

### 3.3. The Rietveld Refinement

**Figure 5:**
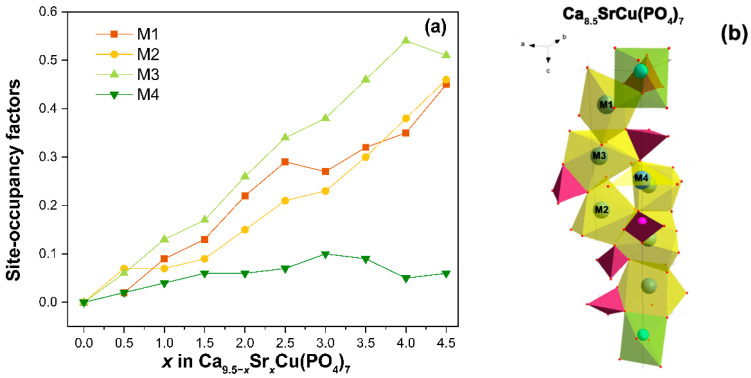
The dependence of the occupation site on the composition Ca_9.5–*x*_Sr*_x_*Cu(PO_4_)_7_ (**a**). The fragment of structure Ca_8.5_SrCu(PO_4_)_7_ (**b**).

Firstly, the occupancies of the crystal sites were refined. Cu^2+^ ions were determined to occupy the smallest octahedral M5 site due to the value of the ionic radii (*r*_VI_ = 0.73 Å). Next, Ca and Sr atoms were randomly placed over the M1–M3 sites. Their occupancies (*a*_i_) were refined with total occupancy restrained to unity. For the M4 site, the occupancy was restricted to a value of 0.5 [7]. According to the refinement, Sr^2+^ with ionic radii (*r*_VIII_ = 1.26 Å) [39] primarily locate in the M1–M3 sites, while in the M4 site, *a*_i_(Sr) does not exceed 0.1 (Figure 5a). Our results are in a good agreement with the literature data from [37], which refined the chemical formula to Ca_9.632_Sr_0.497_Cu_0.371_(PO_4_)_7_ (or β-Ca_2.752_Sr_0.142_Cu_0.106_(PO_4_)_2_), and noted the occupancy of the M4 site by Sr^2+^ ions as 0.110. Further increases in the Sr^2+^ ion concentration results in its distribution through M1–M3, with a preference for the M3 site. This same trend of Sr^2+^ location was observed in the single-doped solid solution Ca_9.5–*x*_Sr*_x_*Cu(PO_4_)_7_ [31].

### 3.4. The Release Behavior of Sr^2+^, Cu^2+^, Ca^2+^ Ions

**Figure 6:**
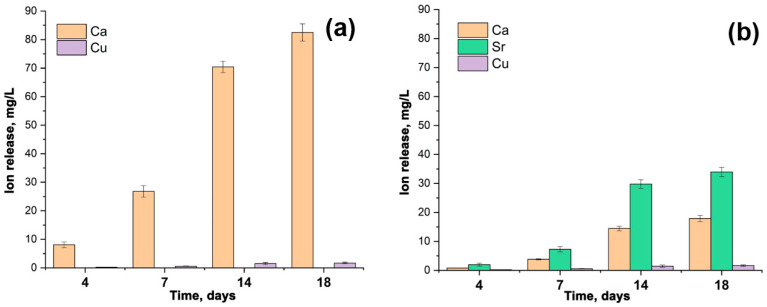
The accumulative release amount of Ca^2+^, Cu^2+^ and Sr^2+^ ions from Ca_9.5_Cu(PO_4_)_7_ (**a**) and Ca_5_Sr_4.5_Cu(PO_4_)_7_ (**b**) samples after soaking in Tris-HCl buffer solution for 4, 7, 14 and 18 days.

The accumulative release amount of Ca^2+^, Cu^2+^, and Sr^2+^ from Ca_9.5–*x*_Sr*_x_*Cu(PO_4_)_7_ samples after soaking in Tris-HCl buffer solution is shown in Figure 6. After 18 days of soaking, the release of Cu^2+^ from the samples exhibits roughly a linear dependency (Figure 6). The amount of Cu^2+^ ion released reaches 1.7 mg/L. There is no significant difference between the Ca_9.5_Cu(PO_4_)_7_ (Figure 6a) and Ca_5_Sr_4.5_Cu(PO_4_)_7_ (Figure 6b) samples in the value of the released Cu^2+^. The notably lower concentrations of Cu^2+^ in the soaking solution, compared to Ca^2+^ and Sr^2+^ ions, are related to its site-selective arrangement in the smallest octahedral M5 site, as proposed by us in [12], and according to crystal structure refinement. The placement of Sr^2+^ in the voluminous M1O_8_–M3O_8_ and M4O_6_ polyhedra (Figure 5b) contributes to its high dissolution into the liquid. The measurements were repeated three times in each step.

### 3.5. Biocompatibility Tests

**Figure 7:**
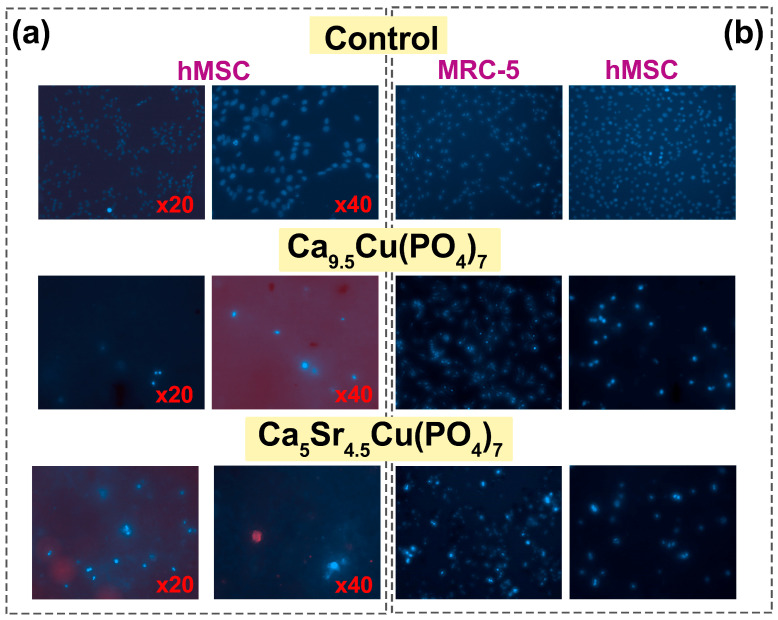
Viability of hMSCs cells after cultivation on a powder layer of Ca_9.5_Cu(PO_4_)_7_ and Ca_5_Sr_4.5_Cu(PO_4_)_7_ samples (**a**). Viability of MRC-5 and hMSCs while adding ceramic powders to cell medium for 3 days (magnification ×20) (**b**).

**Figure 8:**
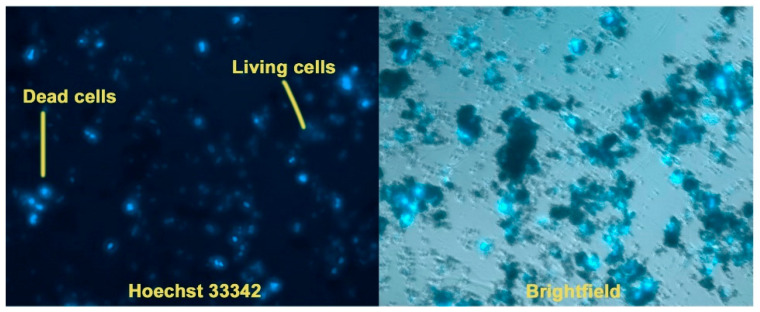
Viability of hMSCs cells after cultivation on a powder layer of Ca_5_Sr_4.5_Cu(PO_4_)_7_ (magnification ×20).

Staining with Hoechst 33342 dye makes it possible to determine the total number of cells, as well as to identify apoptotic cell nuclei. The tests were carried out on samples Ca_9.5_Cu(PO_4_)_7_ and Ca_5_Sr_4.5_Cu(PO_4_)_7_. When using the method of cell cultivation of hMSCs on a powder layer, it was observed that culturing cells on such a layer exhibited a highly cytotoxic effect, evidenced by the presence of individual dead cells after 3 days (Figure 7a). In the second case, it was observed that upon the addition of the studied powders, the proliferative activity significantly decreased, with the morphology of the nuclei indicating complete cell death for the Ca_9.5_Cu(PO_4_)_7_ sample, and partial death for Ca_5_Sr_4.5_Cu(PO_4_)_7_ (Figure 7b). Weakly stained cells with Hoechst 33342 dye remained viable, mainly located on the glass in the gaps between the blocks of powders (Figure 8).

**Figure 9:**
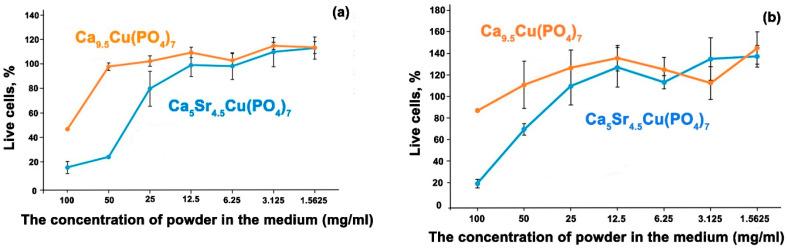
The cytotoxicity of the solution derived from Ca_9.5_Cu(PO_4_)_7_ and Ca_5_Sr_4.5_Cu(PO_4_)_7_ powders on hMSCs cells (**a**), and U-2 OS cells (**b**) assessed using the MTT test.

The MTT test demonstrated high toxicity of aqueous extracts of Ca_5_Sr_4.5_Cu(PO_4_)_7_ powder in maximum concentrations of 25, 50 and 100 mg/mL. Solutions of Ca_9.5_Cu(PO_4_)_7_ powder are less toxic (Figure 9a) even at 100 mg/mL concentration. It is interesting to note that solutions of Ca_9.5_Cu(PO_4_)_7_ exhibit a less toxic effect on osteoblasts, with concentrations exceeding 50 mg/mL showing a stimulating effect on osteoblast proliferation, significantly surpassing control values (Figure 9b). However, the presence of a large quantity of Sr^2+^ ions from the β-TCP structure in Ca_5_Sr_4.5_Cu(PO_4_)_7_ has a negative impact on the cells.

### 3.6. Antibacterial Activity

**Figure 10:**
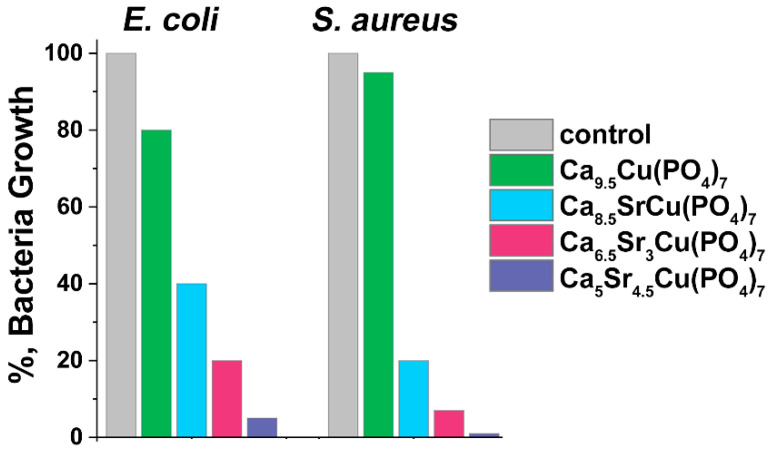
The inhibition of bacteria (*E. coli* and *S. aureus*) grown for 24 h in the presence of Ca_9.5–*x*_Sr*_x_*Cu(PO_4_)_7_. The positive control (ctr) is represented by the growth of each bacteria strain in the absence of phosphates.

Ca_9.5–*x*_Sr*_x_*Cu(PO_4_)_7_ samples showed antibacterial effects against test strains of *E. coli* and *S. aureus* following 24 h of incubation (Figure 10). The study was carried out on the samples where *x* = 0, 1, 3 and 4.5. All samples effectively suppressed bacterial growth. Comparison with our previous study on single doped Ca_10.5–*x*_Cu*_x_*(PO_4_)_7_ solid solutions [12] revealed similar results for bacteria inhibition, particularly for a sample with *x* = 0 (Figure 10). This similarity was also observed in samples Ca_8.5_SrCu(PO_4_)_7_ (or Ca_2.42_Sr_2.86_Cu_2.86_(PO_4_)_2_, Figure 10) and Ca_2.5_Cu_0.25_Sr_0.25_(PO_4_)_2_ [16]. The inhibition of the bacteria growth of approximately 90% on *S. aureus* and 60% on *E. coli* was observed in Ca_2.8_Cu_0.1_Sr_0.1_(PO_4_)_2_ [16].

A significant reduction in bacterial growth was observed when *x* ≥ 1. In our previous study [16], it was suggested that active dopant ions distributed in various crystal sites of the β-TCP structure might augment antimicrobial efficacy. This could be due to the increase in possible concentration of dopant ions and the expansion of the unit cell, leading to a facilitated release of ions. The single-doped Ca_9.5_Cu(PO_4_)_7_ sample inhibits the growth of *E. coli* by 20%, while *S. aureus* shows lower susceptibility to this phosphate with only a 5% inhibition observed (Figure 10). The addition of Sr^2+^ ions to the β-TCP significantly increases its antibacterial activity. For instance, Ca_8.5_SrCu(PO_4_)_7_ inhibits *E. coli* growth by 60%, and *S. aureus* inhibition reaches 80%. A further increase in Sr^2+^ concentration in Ca_9.5–*x*_Sr*_x_*Cu(PO_4_)_7_ results in almost complete suppression of bacterial growth: 93% of inhibition for *E. coli*, and nearly a total reduction in *S. aureus* growth (Figure 10). Notably, according to the results, *S. aureus* is more sensitive to the presence of Sr^2+^ ions in the solution than *E. coli*, and almost indifferent to Cu^2+^ ions. In this study, Ca_9.5–*x*_Sr*_x_*Cu(PO_4_)_7_ exhibited a significant antibacterial activity related to a synergetic effect of Sr^2+^ and Cu^2+^ co-doping.

### 3.7. Piezoelectric Properties

Scanning probe microscopy (SPM) is a commonly used method for investigation of the local piezoelectric properties at the micro(nano)scale, applied for films and (micro)nano particles, and connected to the piezoforce microscopy (PFM) technique. One key challenge in conducting PFM studies on micro(nano) particles is their attachment to a conductive substrate. To address this issue, the particle agglomerates were mixed in isopropyl alcohol and sprayed onto a conductive tape used as the substrate. Through a series of test scans, particles with better adhesion were identified and subsequently utilized for further experiments.

**Figure 11:**
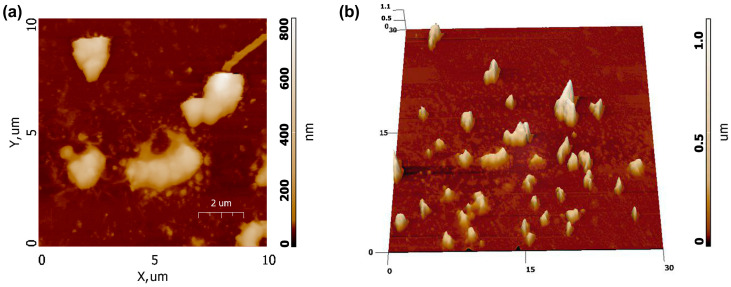
AFM images of Ca_9_Sr_0.5_Cu(PO_4_)_7_ sample: (**a**) 2D AFM map, (**b**) 3D AFM map.

Figure 11 shows AFM scans obtained for a sample prepared according to the procedure described above. As can be seen, the particles of Ca_9_Sr_0.5_Cu(PO_4_)_7_ are inhomogeneously distributed on the substrate and are interconnected into agglomerates, as evident from the 3D scan map (Figure 11b). The linear dimensions of some of the largest particle agglomerates are about 2×3 μm with a height of up to 1 μm.

**Figure 12:**
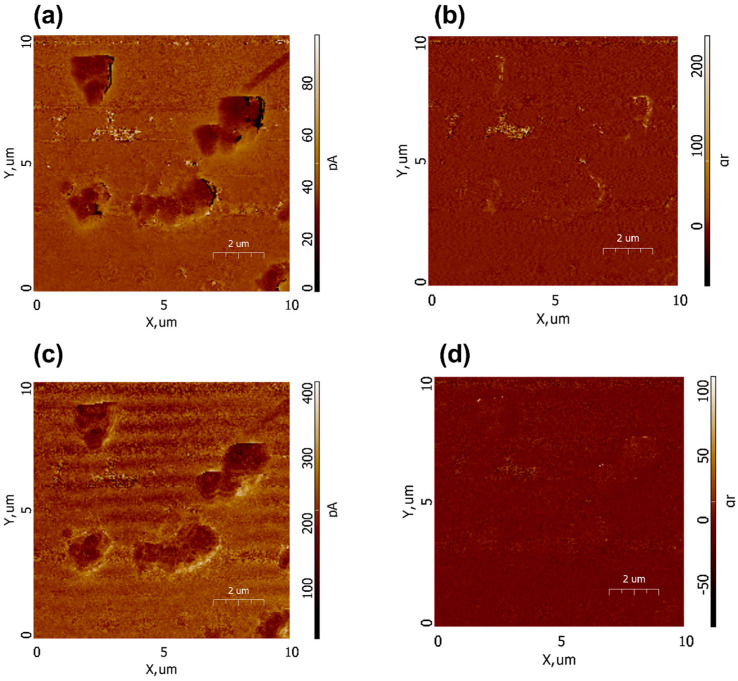
PFM images of Ca_9_Sr_0.5_Cu(PO_4_)_7_ sample: amplitude (**a**,**c**) and phase (**b**,**d**) in vertical (**a**,**b**) and horizontal (**c**,**d**) modes.

To confirm the piezoelectric nature of the Ca_9_Sr_0.5_Cu(PO_4_)_7_, PFM scans of the amplitude and phase in vertical (Figure 12a,b) and horizontal (Figure 12c,d) modes were conducted. A weak piezoresponse was observed on some particles and their agglomerates. The low output signal is also associated with a low amplitude of modulated voltage. The piezoelectric coefficients are proportional to PFM amplitude, and the main challenge in their correct calculations lies in the quantification of PFM signals, which requires calibration. Moreover, the sample demonstrates an «exotic» type of piezoelectricity with the d_14_ piezoelectric coefficient, while more conventional piezoelectric coefficients, such as d_33_ and d_31_, correspond to transverse and longitudinal modes of piezoelectricity. Macroscopic piezoelectricity in calcium phosphates was investigated by S. A. M. Tofail [40], who estimated a shear piezoelectric coefficient of d_14_ = 14pC/N. It should be noted that experiments aimed at directly measuring the macroscopic piezoelectric coefficient d_33_ in Ca_9_Sr_0.5_Cu(PO_4_)_7_ ceramics did not demonstrate piezoelectricity, which may be related to macroscopic anisotropy. However, at micro and nano scales using the PFM technique, piezoelectricity in the sample was observed [41]. For example, the presence of the piezoelectric polarization in tooth enamel was observed by Kalinin in several isolated 50–200 nm regions [42,43]. Finally, the analysis of the obtained results allows for some preliminary conclusions regarding the presence of piezoelectricity in co-doped β-TCP, which necessitates further studies for the accurate quantification of PFM signals to determine piezoelectric coefficients.

## 4. Discussion

In the present study, we demonstrate that in the Ca_9.5–*x*_Sr*_x_*Cu(PO_4_)_7_ solid solution, a continuous series of solid solutions can be formed up to *x* = 4.5 (42.9 mol.% of Sr/(Ca+Sr+Cu)).

In the Ca_10.5–*x*_Sr*_x_*(PO_4_)_7_ series, the limit of isomorphism for β-TCP, upon substitution with strontium ions, was found at *x* = 6. This corresponds to the transition of the structure to the palmierite-type Sr_3_(PO_4_)_2_ [44]. The full incorporation of the Cu^2+^ ions into the M5 site and, consequently, the decrease in the unit cell volume does not affect the isomorphic capacity of the Sr^2+^ ions in Ca_9.5–*x*_Sr*_x_*Cu(PO_4_)_7_. Therefore, within the studied concentration range, a continuous series of solid solutions was confirmed by PXRD and FT-IR studies. The Rietveld refinement revealed that Sr^2+^ and Ca^2+^ ions jointly occupy M1–M4 crystal sites. Strontium prefers the M3 site. The difference in the Sr^2+^ orientation is attributed to the reduced interatomic distances in the M4 polyhedra. In the Ca_9.45_Sr_1.05_(PO_4_)_7_ structure (PDF-4+ № 04-021-3537), the mean distance M4–O (*d*_M4–O_) is 2.622 Å. Conversely, according to the refinement, the *d*_M4–O_ value in Ca_8.5_SrCu(PO_4_)_7_ is 2.386 Å. Given the sum of the ionic radii (*r*_VIII_(Sr^2+^) = 1.26 Å and *r*_II_(O^2–^) = 1.35 Å), the Sr^2+^ location in the M4 site is not suitable for the β-TCP structure due to the nearest oxygen environment is a flat triangle (Figure 5b), which is not loyal to the substitution for large ions. However, the possibility of splitting this position [45] with a change in coordination allows ions with a radius greater than Ca^2+^ to be located there.

Surface morphology significantly influences the adhesion, proliferation and functional activity of cells, including osteoblasts, which play a direct role in osteogenesis. Since the materials in question are powders, their high toxicity is most likely related to physical parameters. When cells are introduced to powders, cell adhesion is disrupted, leading to low survival rates. Conversely, adding powders to cells that are already attached has a significantly less cytotoxic effect. The use of these powders in the form of materials, such as scaffolds, will significantly reduce toxicity [46].

Ca_9.5–*x*_Sr*_x_*Cu(PO_4_)_7_ solid solutions show high antibacterial activity, which can be attributed to the significant release of ions into the solution. The increase in the antibacterial activity of the co-doped CPs [16] was confirmed in the present study. In [16], the inhibition of bacterial growth was reduced due to the presence of the apatite-type phase, known for its lower solubility. The minimal concentration of Sr^2+^ in β-TCP that exhibited effective antibacterial activity was found at *x* = 0.35 in Ca_10.15_Sr_0.35_(PO_4_)_7_ [26]. Importantly, this concentration did not induce cytotoxic effects [47].

Some criteria for enhancing antibacterial activity can be postulated as follows:

(1) Co-doping results in the expansion of the boundaries of single-phase solid solution, thereby enabling the incorporation of a larger number of active ions.
(2) The expansion of the unit cell leads to a more pronounced release of ions into the solution.
(3) Both ions are required to contribute to the antibacterial properties.
(4) The ionic radii should differ significantly to facilitate the preferential localization of co-doped ions at different crystal sites of the host structure.

This study demonstrated that the developed ceramic samples, when incubated in a nutrient medium at 37% under CO_2_ incubator conditions (5% CO_2_, 37 Cº, 95% humidity), in the liquid containing Ca^2+^, Sr^2+^ and Cu^2+^ ions, promoted the active proliferation of osteoblasts. This finding is interesting and will be further investigated, particularly when evaluating solid surfaces obtained from these powders.

Piezoelectric materials have significant potential for various biomedical applications [48]. In the medical field, these materials can function as smart biomaterials that affect cell interactions and biological processes, providing therapy through mechanical stimuli. Charge carriers produced by piezostimulation can catalyze redox reactions and affect biological activity in decontamination, sterilization and therapy processes [49]. Furthermore, studies have shown that piezoelectric signals may affect collagen chemistry or play a direct role in cellular activity [50,51]. β-TCP-based materials are promising candidates for biological applications due to their excellent biocompatibility, bone formation characteristics and cell adhesion ability, as well as their piezocatalytic properties [52].

To enhance piezoelectric properties, polyvinylidene fluoride (PVDF) coatings [53], composites [54] and scaffolds [55] are often employed. These materials have demonstrated positive effects on osteogenesis and cell proliferation [56] as well as bone treatment [57]. In the Ca_9_Sr_0.5_Cu(PO_4_)_7_ sample, a weak piezoelectric response was observed. Previously, Mg^2+^-doped β-TCP phosphates [55,58] were studied. Piezoelectric materials are characterized by crystal structures that lack central symmetry. In the case of hydroxyapatite, polar and noncentrosymmetric structures arise due to ferroelectric ordering of hydroxyl ions (OH) along the crystallographic c axis (direction [001]) [52] The enhancement of piezoelectricity in phosphates can be attributed to several factors, including the crystal structure and symmetry, the presence of certain ions or defects, electric polarization and mechanical stress. Additionally, the alignment and rotation of the phosphate groups (PO_4_) within the crystal structure can significantly influence the piezoelectric behavior. Another important aspect is the charge distribution within the phosphate groups. The uneven distribution of charge can lead to an electric dipole moment, which is a key factor in piezoelectricity. The piezoelectric properties can also be influenced by the presence of impurities or doped ions in the crystal lattice. Numerous studies have explored the impact of doping on the piezoelectric properties of materials [59]. These impurities can perturb the regular arrangement of atoms and create local regions of polarization, enhancing the overall piezoelectric response.

The incorporation of Sr and Cu ions leads to a certain shift in the electron density in the crystal sites due to the difference in the electronegativity (χ) of Ca^2+^ (χ = 1.00) host ions and Cu (χ = 1.9). The increase in the difference in the electronegativity value can contribute to the piezoelectric properties. It is important to note that the piezoelectric properties of the material are affected not only by the electronegativity of the substituted atoms but also by their ionic radius. For instance, isovalent substitution of Ca^2+^ ions (r_VI_ = 1.00 Å) with larger Sr^2+^ ions (r_VI_ = 1.18 Å) [39] can affect the properties. The absence of second-order phase transitions, combined with chemical pressure, leads to lattice distortion and the emergence of an additional dipole moment.

## 5. Conclusions

Multifunctional biomimetic co-doped Ca_9.5–*x*_Sr*_x_*Cu(PO_4_)_7_ solid solutions were synthesized in the powder form by solid-state synthesis. Up to *x* =4.5 in Ca_9.5–*x*_Sr*_x_*Cu(PO_4_)_7_, structural saturation by Sr^2+^ ions was not observed, as confirmed by PXRD studies. A very small amount of pyrophosphate impurity detected by the FT-IR method was found only in the β-Ca_3_(PO_4_)_2_ sample. The Rietveld refinement revealed that Sr^2+^ ions are distributed among M1–M4 sites, while M5 is fully occupied by Cu^2+^. The biological study showed a dose-dependent effect on the hMSCs and U-2 OS cell lines. At concentration less than 25 mg/mL, a positive effect on the cells’ proliferation was observed. A significant antibacterial effect was detected against *E. coli* and *S. aureus*, which is associated with the synergetic effect of co-doping with Sr^2+^ and Cu^2+^ releases into the solution. Solid solutions Ca_9.5–*x*_Sr*_x_*Cu(PO_4_)_7_ with *x* ≤ 3 can be used as bone substitutes. The presence of the piezoelectric response contributes to confer biomimetic properties to Ca_9.5–*x*_Sr*_x_*Cu(PO_4_)_7_ material.

## Figures and Tables

**Table 1:** Chemical formula, doping mol.%, unit cell (*a*, *c*) parameters and volume (*V*) in Ca_9.5–*x*_Sr*_x_*Cu(PO_4_)_7_ 0 ≤ *x* ≤ 4.5 samples.

Chemical Formula	mol.%, Sr^2+^	*a*, Å	*c*, Å	*V*, Å^3^
Ca_9.5_Cu(PO_4_)_7_	0	10.3430(1)	37.226(5)	3448.8(5)
Ca_9_Sr_0.5_Cu(PO_4_)_7_	4.8	10.3631(7)	37.302(3)	3469.4(3)
Ca_8.5_SrCu(PO_4_)_7_	9.5	10.3882(9)	37.421(4)	3497.3(4)
Ca_8_Sr_1.5_Cu(PO_4_)_7_	14.3	10.4101(3)	37.518(1)	3521.4(8)
Ca_7.5_Sr_2_Cu(PO_4_)_7_	19.0	10.4281(2)	37.633(7)	3544.1(7)
Ca_7_Sr_2.5_Cu(PO_4_)_7_	23.8	10.4501(4)	37.780(5)	3578.1(5)
Ca_6.5_Sr_3_Cu(PO_4_)_7_	28.6	10.4671(4)	37.927(7)	3591.7(7)
Ca_6_Sr_3.5_Cu(PO_4_)_7_	33.3	10.4842(2)	38.092(6)	3626.1(7)
Ca_5.5_Sr_4_Cu(PO_4_)_7_	38.1	10.5003(2)	38.243(6)	3651.6(7)
Ca_5_Sr_4.5_Cu(PO_4_)_7_	42.8	10.5210(4)	38.414(3)	3682.7(5)

## Data Availability

The research data are available upon an official reasonable request.

## Supplementary Materials

The following supporting information can be downloaded at: https://www.mdpi.com/article/10.3390/biomimetics9040252/s1, Figure S1: The dependence of the unit cell parameters a and c for synthesized solid solutions Ca_9.5–*x*_Sr*_x_*Cu(PO_4_)_7_; Intensity profiles for the powder X-ray Rietveld refinement of Figure S2: Ca_9.5_Cu(PO_4_)_7_; Figure S3: Ca_9_Sr_0.5_Cu(PO_4_)_7_; Figure S4: Ca_8.5_SrCu(PO_4_)_7_; Figure S5: Ca_8_Sr_1.5_Cu(PO_4_)_7_; Figure S6: Ca_7.5_Sr_2_Cu(PO_4_)_7_; Figure S7: Ca_7_Sr_2.5_Cu(PO_4_)_7_; Figure S8: Ca_6.5_Sr_3_Cu(PO_4_)_7_; Figure S9: Ca_6_Sr_3.5_Cu(PO_4_)_7_; Figure S10: Ca_5.5_Sr_4_Cu(PO_4_)_7_; Figure S11: Intensity profiles for the powder X-ray Rietveld refinement of Ca_5_Sr_4.5_Cu(PO_4_)_7_; Table S1: Chemical formula, sample code, unit cell (*a*, *c*) parameters and volume (*V*) in Ca_9.5–*x*_Sr*_x_*Cu(PO_4_)_7_ 0 ≤ *x* ≤ 4.5 samples; Table S2: Main crystallographic and experimental data on Ca_9.5–*x*_Sr*_x_*Cu(PO_4_)_7_ (2.5 ≤ *x* ≤ 4.5); Atomic coordinates, displacement parameters (Å^2^) and site-occupancy factors (SOFs) in the structure of Table S3: Ca_9.5_Cu(PO_4_)_7_; Table S4: Ca_9_Sr_0.5_Cu(PO_4_)_7_; Table S5: Ca_8.5_SrCu(PO_4_)_7_; Table S6: Ca_8_Sr_1.5_Cu(PO_4_)_7_; Table S7: Ca_7.5_Sr_2_Cu(PO_4_)_7_; Table S8: Ca_7_Sr_2.5_Cu(PO_4_)_7_; Table S9: Ca_6.5_Sr_3.5_Cu(PO_4_)_7_; Table S10: Ca_6_Sr_3.5_Cu(PO_4_)_7_; Table S11: Ca_5.5_Sr_4_Cu(PO_4_)_7_; Table S12: Ca_5_Sr_4.5_Cu(PO_4_)_7_.
