# Supplementary material for: Strontium and Copper Co-Doped Multifunctional Calcium Phosphates: Biomimetic and Antibacterial Materials for Bone Implants
Source: Biomimetics (Basel). 2024 Apr 20;9(4):252. doi: 10.3390/biomimetics9040252 (PMC11048597; doi:10.3390/biomimetics9040252)
Supplement: Supplementary file 1 [file biomimetics-09-00252-s001.zip › biomimetics-2942313-supplementary.pdf]

Supporting Information

Strontium and Copper Co-Doped Multifunctional Calcium Phosphates: Biomimetic and Antibacterial Materials for Bone Implants

Vladimir N. Lebedev [1], Mariya I. Kharovskaya [1], Bogdan I. Lazoryak [1], Anastasiya O. Solovieva [2], Inna V. Fadeeva [3], Abdulkarim A. Amirov [4], Maksim A. Koliushenkov [5], Farid F. Orudzhev [6], Oksana V. Baryshnikova [1], Viktoriya G. Yankova [7], Julietta V. Rau [7,8] and Dina V. Deyneko [1,9,*]

[1] Chemistry Department, Lomonosov Moscow State University, Leninskie Gory 1, 119991 Moscow, Russia; vladimir.lebedev@chemistry.msu.ru (V.N.L.); masha.harovskaaya@gmail.com (M.I.K.); bilazoryak@gmail.com (B.I.L.); sheoksana@yandex.ru (O.V.B.)

[2] Laboratory of Pharmacology Active Compounds, Research Institute of Clinical and Experimental Lymphology–Branch of the Institute of Cytology and Genetics, Siberian Branch of Russian Academy of Sciences (RICEL–Branch of IC&G SB RAS), 630060 Novosibirsk, Russia; solovevaao@gmail.com

[3] A.A. Baikov Institute of Metallurgy and Material Science RAS, Leninskie, 49, 119334 Moscow, Russia; fadeeva_inna@mail.ru

[4] Amirkhanov Institute of Physics, Dagestan Scientific Center of Russian Academy of Sciences, 367003 Makhachkala, Russia; amiroff_a@mail.ru

[5] Physics Department, Lomonosov Moscow State University, Leninskie Gori 1, 119991 Moscow, Russia; koliushenkov.ma19@physics.msu.ru

[6] Geothermal and Renewal Energy Institute of the High Temperature Joint Institute of the Russian Academy of Sciences, 367015 Makhachkala, Russia; farid-stkha@mail.ru

[7] Institute of Pharmacy, Department of Analytical, Physical and Colloid Chemistry, I.M. Sechenov First Moscow State Medical University, Trubetskaya 8, building 2, 119048 Moscow, Russia; yankova_v_g@staff.sechenov.ru (V.G.Y.); giulietta.rau@ism.cnr.it (J.V.R.)

[8] Istituto di Struttura della Materia, Consiglio Nazionale delle Ricerche, ISM-CNR, Via del Fosso del Cavaliere 100, 00133 Rome, Italy

[9] Laboratory of Arctic Mineralogy and Material Sciences, Kola Science Centre RAS, 14 Fersman Str.,184209 Apatity, Russia

* Correspondence: deynekomsu@gmail.com

Table S1. Chemical formula, sample code, unit cell (*a, c*) parameters and volume (*V*) in $Ca_{9.5-x}Sr_xCu(PO_4)_7$ $0 \leq x \leq 4.5$ samples.

**Chemical formula**	***x*, Sr^{2+}**	**mol.%, Sr^{2+}**	***a*, Å**	***c*, Å**	***V*, Å**
$Ca_{9.5}Cu(PO_4)_7$	0	0	10.3430(1)	37.226(5)	3448.8(5)
$Ca_9Sr_{0.5}Cu(PO_4)_7$	0.5	5.556	10.3631(7)	37.302(3)	3469.4(3)
$Ca_{8.5}SrCu(PO_4)_7$	1	11.765	10.3882(9)	37.421(4)	3497.3(4)
$Ca_8Sr_{1.5}Cu(PO_4)_7$	1.5	18.750	10.4101(3)	37.518(1)	3521.4(8)
$Ca_{7.5}Sr_2Cu(PO_4)_7$	2	26.667	10.4281(2)	37.633(7)	3544.1(7)
$Ca_7Sr_{2.5}Cu(PO_4)_7$	2.5	35.714	10.4501(4)	37.780(5)	3578.1(5)
$Ca_{6.5}Sr_3Cu(PO_4)_7$	3	46.154	10.4671(4)	37.927(7)	3591.7(7)
$Ca_6Sr_{3.5}Cu(PO_4)_7$	3.5	58.333	10.4842(2)	38.092(6)	3626.1(7)
$Ca_{5.5}Sr_4Cu(PO_4)_7$	4	72.727	10.5003(2)	38.243(6)	3651.6(7)
$Ca_5Sr_{4.5}Cu(PO_4)_7$	4.5	90.000	10.5210(4)	38.414(3)	3682.7(5)

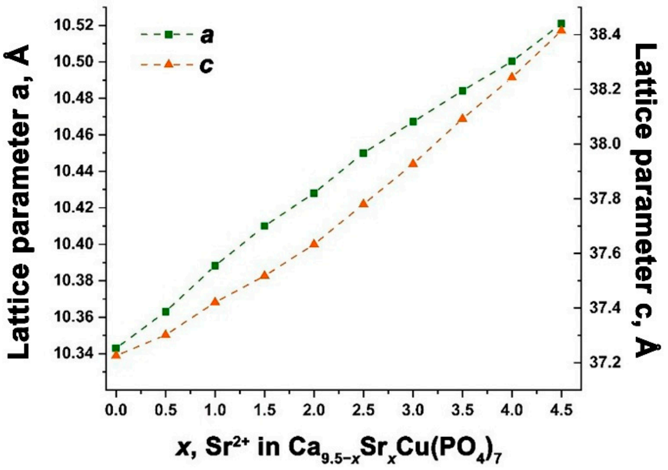

Figure S1. The dependence of the unit cell parameters *a* and *c* for synthesized solid solutions $Ca_{9.5-x}Sr_xCu(PO_4)_7$

Table S2. Main crystallographic and experimental data on $Ca_{9.5-x}Sr_xCu(PO_4)_7$ $(2.5 \leq x \leq 4.5)$.

Sample	$Ca_7Sr_{2.5}Cu(PO_4)_7$	$Ca_{6.5}Sr_3Cu(PO_4)_7$	$Ca_6Sr_{3.5}Cu(PO_4)_7$	$Ca_{5.5}Sr_{4.0}Cu(PO_4)_7$	$Ca_5Sr_{4.5}Cu(PO_4)_7$
Formula from Rietveld Refinement	$Ca_{7.15}Sr_{2.35}Cu(PO_4)_7$	$Ca_{6.828}Sr_{2.702}Cu(PO_4)_7$	$Ca_{6.216}Sr_{3.284}Cu(PO_4)_7$	$Ca_{5.629}Sr_{3.871}Cu(PO_4)_7$	$Ca_{5.163}Sr_{4.337}Cu(PO_4)_7$

M_r	1220.83	1238.78	1265.21	1293.12	1315.28
Temperature, K	293				
Crystal system, space group	Trigonal, R3c				
Radiation type	Cu *Kα*				
Diffractometer	Rigaku SmartLab SE				
θ-Range	3.000- 90.000, step size (°) 0.02				
D_x	3.3941	3.4416	3.4892	3.5439	3.5788
R_p	7.27	6.87	5.86	5.55	5.63
R_{wp}	9.97	9.22	7.79	7.35	7.44
R_{Bragg}	4.39	4.18	4.15	4.19	4.13
Goodness of fit (ChiQ)	2.27	2.21	1.88	1.75	1.80
Max./min. residual density (e×Å^{-3})	1.37/-2.10	2.22/-3.50	1.39/-3.12	1.23/-2.13	1.53/-2.01
No. of parameters	62	63	62	65	62

Table S3. Atomic coordinates, displacement parameters (Å^2) and site-occupancy factors (SOFs) in the structure of $Ca_{9.5}Cu(PO_4)_7$.

Atom	Wyckoff site	*x*	*y*	*z*	U_{iso}, Å^2	SOF
M1	18*b*	0.7259	0.8576	0.4316	0.0084	$Ca_{1.0}$
M2	18*b*	0.6183	0.8226	0.2306	0.0084	$Ca_{1.0}$
M3	18*b*	0.1249	0.2721	0.3252	0.0084	$Ca_{1.0}$
M4	6*a*	0	0	0.1819	0.0084	$Cu_{1.0}$
M5	6*a*	0	0	0	0.0122	$Cu_{1.0}$
P1	6*a*	0	0	0.2639	0.0165	$P_{1.0}$
P2	18*b*	0.6844	0.8595	0.134	0.0099	$P_{1.0}$
P3	18*b*	0.6515	0.8437	0.0307	0.0086	$P_{1.0}$
O1	6*a*	0	0	0.3061	0.0089	$O_{1.0}$
O2	6*a*	0.0161	0.8675	0.2544	0.0089	$O_{1.0}$
O3	18*b*	0.734	0.916	0.1736	0.0089	$O_{1.0}$

O4	18*b*	0.762	0.778	0.122	0.0089	$O_{1.0}$
O5	18*b*	0.724	0.006	0.1131	0.0089	$O_{1.0}$
O6	6*a*	0.513	0.758	0.1318	0.0089	$O_{1.0}$
O7	18*b*	0.604	0.955	0.045	0.0089	$O_{1.0}$
O8	18*b*	0.574	0.691	0.0519	0.0089	$O_{1.0}$
O9	18*b*	0.825	0.922	0.0403	0.0089	$O_{1.0}$
O10	18*b*	0.6243	0.8246	0.9916	0.0089	$O_{1.0}$

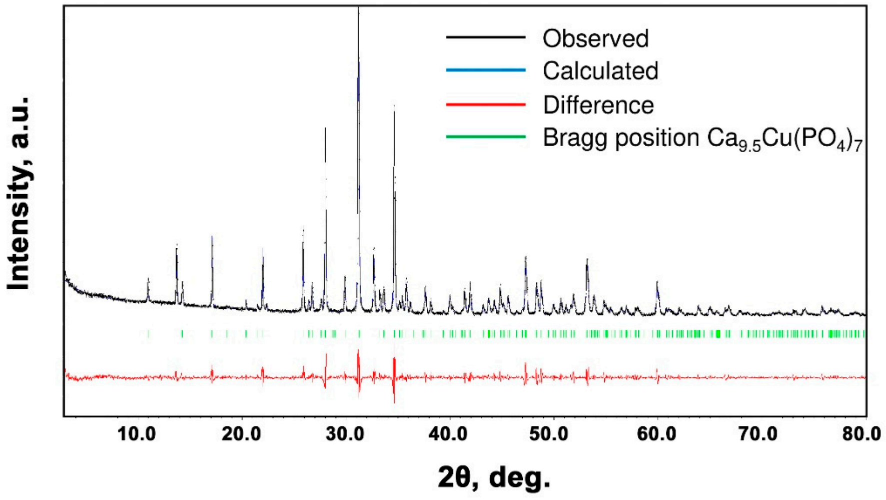

Figure S2. Intensity profiles for the powder X-ray Rietveld refinement of $Ca_{9.5}Cu(PO_4)_7$. The observed and calculated profiles are represented in black and blue lines, respectively. The difference in the profile is plotted at the bottom (red line). Vertical bars indicate the positions of the Bragg reflections (green bars).

Table S4. Atomic coordinates, displacement parameters (Å^2) and site-occupancy factors (SOFs) in the structure of $Ca_9Sr_{0.5}Cu(PO_4)_7$.

Atom	Wyckoff site	*x*	*y*	*z*	U_{iso}, Å^2	SOF
M1	18*b*	0.7259(2)	0.8582(6)	0.4356(3)	0.0097(2)	$Ca_{0.98}$+$Sr_{0.02}$
M2	18*b*	0.6213(3)	0.8193(9)	0.2337(3)	0.015(9)	$Ca_{0.93}$+$Sr_{0.07}$
M3	18*b*	0.1258(3)	0.2746(9)	0.3295(3)	0.0048(2)	$Ca_{0.94}$+$Sr_{0.06}$
M4	6*a*	0	0	0.1777(6)	0.005(3)	$Ca_{0.98}$+$Sr_{0.02}$
M5	6*a*	0	0	0.0034(5)	0.0473(3)	$Cu_{1.0}$
P1	6*a*	0	0	0.2607(8)	0.059(1)	$P_{1.0}$
P2	18*b*	0.6891(13)	0.859(2)	0.1376(5)	0.0095(1)	$P_{1.0}$
P3	18*b*	0.6523(17)	0.851(2)	0.0351(5)	0.0024(1)	$P_{1.0}$
O1	6*a*	0	0	0.3020(8)	0.0089	$O_{1.0}$
O2	6*a*	0.035(3)	0.868(3)	0.2583(1)	0.0089	$O_{1.0}$
O3	18*b*	0.739(4)	0.924(3)	0.1755(5)	0.0089	$O_{1.0}$
O4	18*b*	0.762(5)	0.768(4)	0.1267(9)	0.0089	$O_{1.0}$
O5	18*b*	0.728(5)	0.002(4)	0.1169(9)	0.0089	$O_{1.0}$
O6	6*a*	0.5180(5)	0.761(5)	0.1363(1)	0.0089	$O_{1.0}$
O7	18*b*	0.609(4)	0.965(3)	0.0485(1)	0.0089	$O_{1.0}$
O8	18*b*	0.571(4)	0.696(3)	0.0530(1)	0.0089	$O_{1.0}$
O9	18*b*	0.818(2)	0.929(4)	0.0464(1)	0.0089	$O_{1.0}$
O10	18*b*	0.613(3)	0.811(5)	0.9945(7)	0.0089	$O_{1.0}$

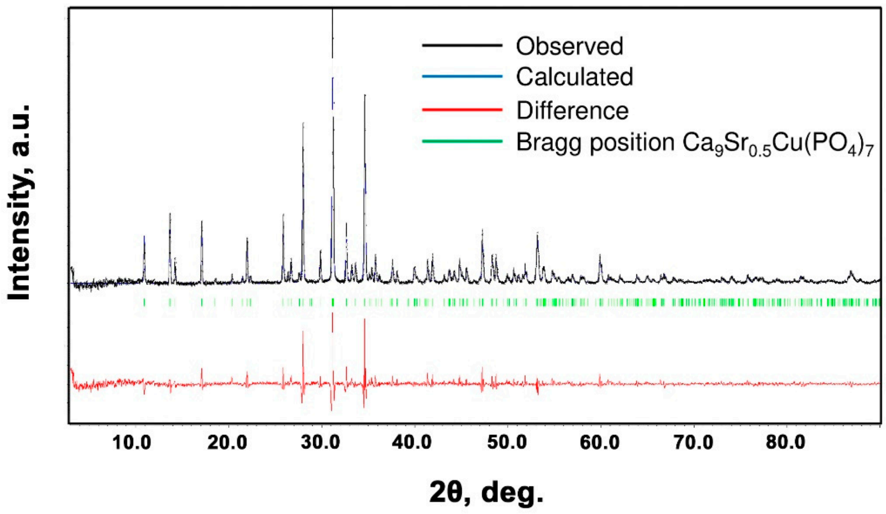

Figure S3. Intensity profiles for the powder X-ray Rietveld refinement of $Ca_9Sr_{0.5}Cu(PO_4)_7$. The observed and calculated profiles are represented in black and blue lines, respectively. The difference in the profile is plotted at the bottom (red line). Vertical bars indicate the positions of the Bragg reflections (green bars).

Table S5. Atomic coordinates, displacement parameters (Å^2) and site-occupancy factors (SOFs) in the structure of $Ca_{8.5}SrCu(PO_4)_7$.

Atom	Wyckoff site	*x*	*y*	*z*	U_{iso}, Å^2	SOF
M1	18*b*	0.7266(8)	0.8558(2)	0.43561(8)	0.0154(3)	$Ca_{0.91}$+$Sr_{0.09}$
M2	18*b*	0.6253(9)	0.8195(2)	0.2329(2)	0.0161(8)	$Ca_{0.93}$+$Sr_{0.07}$
M3	18*b*	0.1351(3)	0.2779(7)	0.33057(9)	0.016(8)	$Ca_{0.87}$+$Sr_{0.13}$
M4	6*a*	0	0	0.1807(4)	0.0031(1)	$Ca_{0.96}$+$Sr_{0.04}$
M5	6*a*	0	0	0.0002(4)	0.0583(1k)	$Cu_{1.0}$
P1	6*a*	0	0	0.2592(6)	0.048(8)	$P_{1.0}$
P2	18*b*	0.6993(3)	0.844(2)	0.1374(5)	0.071(6)	$P_{1.0}$
P3	18*b*	0.6534(2)	0.8515(2)	0.0329(4)	0.013(3)	$P_{1.0}$
O1	6*a*	0	0	0.3004(6)	0.0089	$O_{1.0}$
O2	6*a*	0.033(2)	0.878(2)	0.2596(7)	0.0089	$O_{1.0}$
O3	18*b*	0.742(2)	0.917(2)	0.1747(5)	0.0089	$O_{1.0}$

O4	18*b*	0.773(3)	0.755(3)	0.1245(7)	0.0089	$O_{1.0}$
O5	18*b*	0.721(3)	-0.007(3)	0.1205(6)	0.0089	$O_{1.0}$
O6	6*a*	0.5295(4)	0.779(4)	0.1375(7)	0.0089	$O_{1.0}$
O7	18*b*	0.614(3)	0.964(2)	0.0491(8)	0.0089	$O_{1.0}$
O8	18*b*	0.570(2)	0.7090(18)	0.0555(5)	0.0089	$O_{1.0}$
O9	18*b*	0.8103(15)	0.923(3)	0.0494(6)	0.0089	$O_{1.0}$
O10	18*b*	0.614(2)	0.822(3)	0.9998(6)	0.0089	$O_{1.0}$

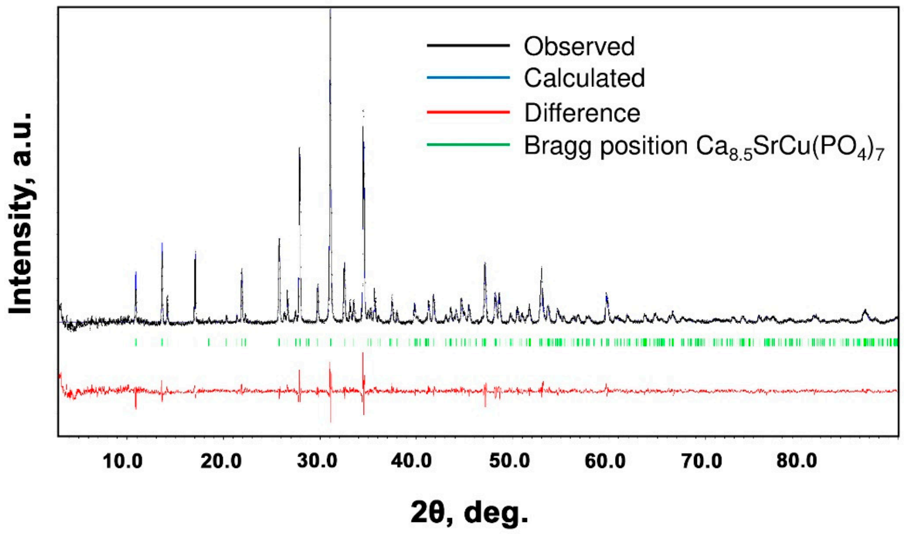

Figure S4. Intensity profiles for the powder X-ray Rietveld refinement of $Ca_{8.5}SrCu(PO_4)_7$. The observed and calculated profiles are represented in black and blue lines, respectively. The difference in the profile is plotted at the bottom (red line). Vertical bars indicate the positions of the Bragg reflections (green bars).

Table S6. Atomic coordinates, displacement parameters (Å^2) and site-occupancy factors (SOFs) in the structure of $Ca_8Sr_{1.5}Cu(PO_4)_7$.

Atom	Wyckoff site	*x*	*y*	*z*	U_{iso}, Å^2	SOF
M1	18*b*	0.7232(9)	0.8582(4)	0.4342(2)	0.0095(2)	$Ca_{0.87}$+$Sr_{0.13}$
M2	18*b*	0.6230(1)	0.8190(7)	0.2321(2)	0.0185(8)	$Ca_{0.91}$+$Sr_{0.09}$
M3	18*b*	0.1346(3)	0.2830(7)	0.3276(3)	0.0273(3)	$Ca_{0.83}$+$Sr_{0.17}$
M4	6*a*	0	0	0.1808(5)	0.0026(1)	$Ca_{0.94}$+$Sr_{0.06}$

M5	6*a*	0	0	0.0024(6)	0.0961(1)	$Cu_{1.0}$
P1	6*a*	0	0	0.2601(6)	0.035(8)	$P_{1.0}$
P2	18*b*	0.6884(3)	0.8685(8)	0.1340(5)	0.012(5)	$P_{1.0}$
P3	18*b*	0.6483(6)	0.836(2)	0.0330(5)	0.023(5)	$P_{1.0}$
O1	6*a*	0	0	0.3012(6)	0.0089	$O_{1.0}$
O2	6*a*	0.004(2)	0.864(2)	0.2403(7)	0.0089	$O_{1.0}$
O3	18*b*	0.747(2)	0.905(3)	0.1725(5)	0.0089	$O_{1.0}$
O4	18*b*	0.760(4)	0.775(3)	0.1230(7)	0.0089	$O_{1.0}$
O5	18*b*	0.740(4)	0.017(3)	0.1155(7)	0.0089	$O_{1.0}$
O6	6*a*	0.5224(6)	0.750(3)	0.1341(8)	0.0089	$O_{1.0}$
O7	18*b*	0.607(4)	0.952(3)	0.0453(8)	0.0089	$O_{1.0}$
O8	18*b*	0.570(3)	0.690(3)	0.0544(7)	0.0089	$O_{1.0}$
O9	18*b*	0.8161(7)	0.923(3)	0.0411(8)	0.0089	$O_{1.0}$
O10	18*b*	0.626(2)	0.825(4)	0.9973(6)	0.0089	$O_{1.0}$

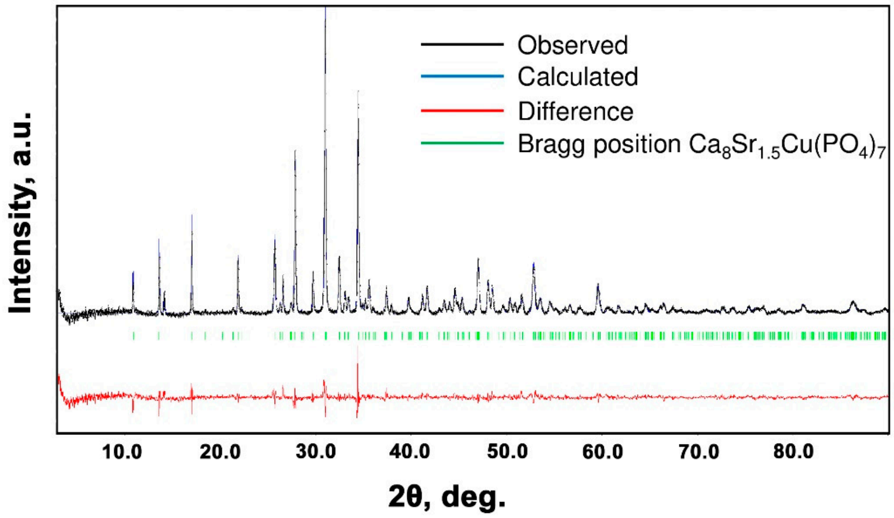

Figure S5. Intensity profiles for the powder X-ray Rietveld refinement of $Ca_8Sr_{1.5}Cu(PO_4)_7$. The observed and calculated profiles are represented in black and blue lines, respectively. The

difference in the profile is plotted at the bottom (red line). Vertical bars indicate the positions of the Bragg reflections (green bars).

Table S7. Atomic coordinates, displacement parameters (Å^2) and site-occupancy factors (SOFs) in the structure of $Ca_{7.5}Sr_2Cu(PO_4)_7$.

Atom	Wyckoff site	*x*	*y*	*z*	U_{iso}, Å^2	SOF
M1	18*b*	0.7198(3)	0.8545(6)	0.4363(3)	0.0172(9)	$Ca_{0.78}$+$Sr_{0.22}$
M2	18*b*	0.6269(8)	0.810(2)	0.2337(3)	0.0535(5)	$Ca_{0.85}$+$Sr_{0.15}$
M3	18*b*	0.1617(6)	0.2908(3)	0.2908(3)	0.0299(3)	$Ca_{0.74}$+$Sr_{0.26}$
M4	6*a*	0	0	0.1810(8)	0.0015(1)	$Ca_{0.94}$+$Sr_{0.06}$
M5	6*a*	0	0	0.0017(7)	0.0508(2)	$Cu_{1.0}$
P1	6*a*	0	0	0.2483(1)	0.0144(8)	$P_{1.0}$
P2	18*b*	0.6862(7)	0.840(2)	0.1373(5)	0.0117(4)	$P_{1.0}$
P3	18*b*	0.644(3)	0.852(3)	0.0364(7)	0.0353(3)	$P_{1.0}$
O1	6*a*	0	0)	0.2893(1)	0.0089	$O_{1.0}$
O2	6*a*	0.002(4)	0.852(4)	0.2317(1)	0.0089	$O_{1.0}$
O3	18*b*	0.733(4)	0.895(4)	0.1757(6)	0.0089	$O_{1.0}$
O4	18*b*	0.769(4)	0.770(4)	0.1193(1)	0.0089	$O_{1.0}$
O5	18*b*	0.733(5)	0.013(5)	0.1116(1)	0.0089	$O_{1.0}$
O6	6*a*	0.524(2)	0.766(5)	0.1242(1)	0.0089	$O_{1.0}$
O7	18*b*	0.548(4)	0.918(5)	0.0240(2)	0.0089	$O_{1.0}$
O8	18*b*	0.582(5)	0.698(3)	0.0534(1)	0.0089	$O_{1.0}$
O9	18*b*	0.807(2)	0.892(5)	0.0319(1)	0.0089	$O_{1.0}$
O10	18*b*	0.626(4)	0.768(4)	0.9966(1)	0.0089	$O_{1.0}$

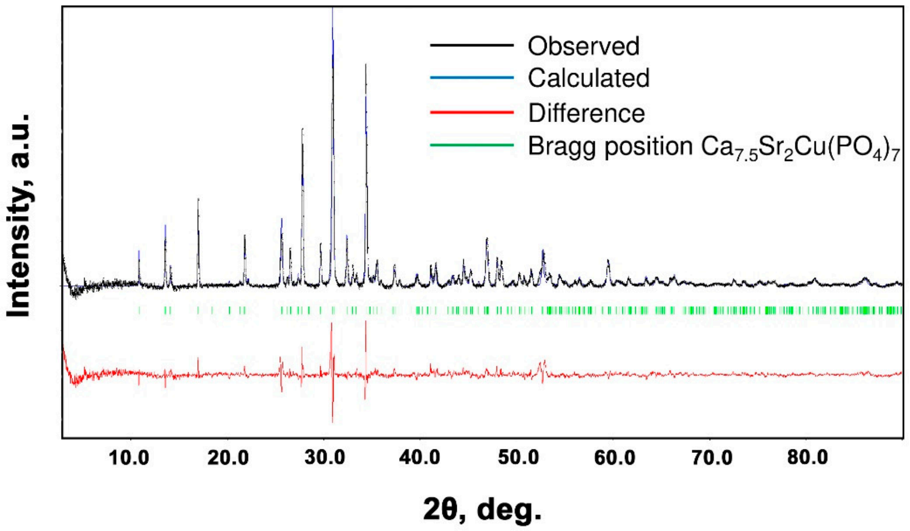

Figure S6. Intensity profiles for the powder X-ray Rietveld refinement of $Ca_{7.5}Sr_2Cu(PO_4)_7$. The observed and calculated profiles are represented in black and blue lines, respectively. The difference in the profile is plotted at the bottom (red line). Vertical bars indicate the positions of the Bragg reflections (green bars).

Table S8. Atomic coordinates, displacement parameters (Å^2) and site-occupancy factors (SOFs) in the structure of $Ca_7Sr_{2.5}Cu(PO_4)_7$.

Atom	Wyckoff site	*x*	*y*	*z*	U_{iso}, Å^2	SOF
M1	18*b*	0.7261(2)	0.8579(4)	0.4365(3)	0.0232(7)	$Ca_{0.71}$+$Sr_{0.29}$
M2	18*b*	0.6313(0)	0.8126(7)	0.2354(3)	0.0035(8)	$Ca_{0.79}$+$Sr_{0.21}$
M3	18*b*	0.1468(6)	0.2859(1)	0.3329(3)	0.0318(2)	$Ca_{0.66}$+$Sr_{0.34}$
M4	6*a*	0	0	0.1761(9)	0.0314(2)	$Ca_{0.93}$+$Sr_{0.07}$
M5	6*a*	0	0	-0.0036(5)	0.0122(1)	$Cu_{1.0}$
P1	6*a*	0	0	0.2601(9)	0.0127(9)	$P_{1.0}$
P2	18*b*	0.6870(6)	0.838(2)	0.1324(6)	0.0089(4)	$P_{1.0}$
P3	18*b*	0.658(2)	0.819(3)	0.0322(5)	0.0031(4)	$P_{1.0}$
O1	6*a*	0	0	0.3008(9)	0.0089	$O_{1.0}$
O2	6*a*	-0.023(4)	0.106(4)	0.2363(8)	0.0089	$O_{1.0}$
O3	18*b*	0.731(4)	0.922(4)	0.1679(6)	0.0089	$O_{1.0}$

O4	18*b*	0.780(4)	0.796(4)	0.1086(9)	0.0089	$O_{1.0}$
O5	18*b*	0.775(5)	0.755(4)	0.1322(1)	0.0089	$O_{1.0}$
O6	6*a*	0.557(3)	0.868(4)	0.1289(1)	0.0089	$O_{1.0}$
O7	18*b*	0.629(4)	0.939(4)	0.0492(1)	0.0089	$O_{1.0}$
O8	18*b*	0.593(4)	0.671(3)	0.0524(1)	0.0089	$O_{1.0}$
O9	18*b*	0.813(2)	0.940(4)	0.0438(1)	0.0089	$O_{1.0}$
O10	18*b*	0.618(3)	0.819(4)	0.9940(9)	0.0089	$O_{1.0}$

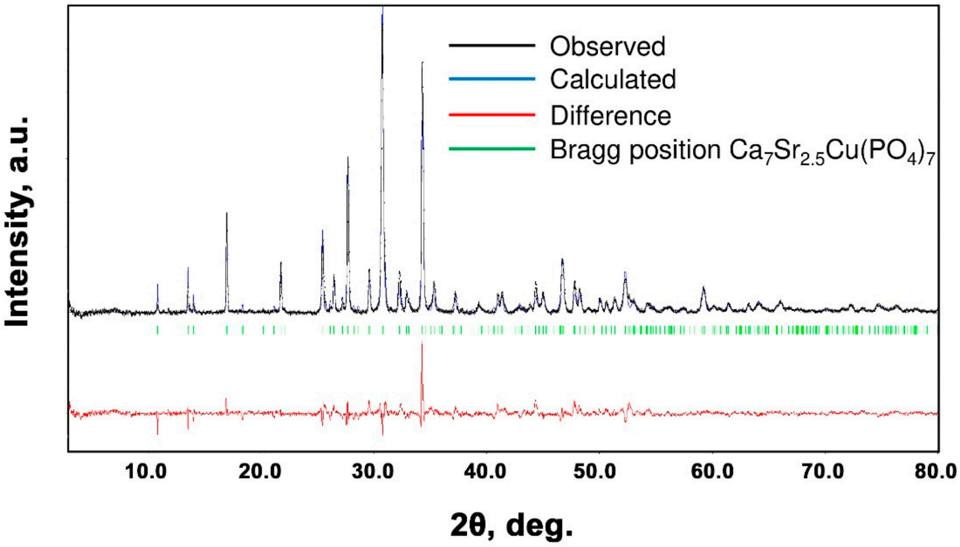

Figure S7. Intensity profiles for the powder X-ray Rietveld refinement of $Ca_7Sr_{2.5}Cu(PO_4)_7$. The observed and calculated profiles are represented in black and blue lines, respectively. The difference in the profile is plotted at the bottom (red line). Vertical bars indicate the positions of the Bragg reflections (green bars).

Table S9. Atomic coordinates, displacement parameters (Å^2) and site-occupancy factors (SOFs) in the structure of $Ca_{6.5}Sr_{3.5}Cu(PO_4)_7$.

Atom	Wyckoff site	*x*	*y*	*z*	U_{iso}, Å^2	SOF
M1	18*b*	0.7207(3)	0.8595(6)	0.4341(3)	0.0087(1)	$Ca_{0.73}$+$Sr_{0.27}$
M2	18*b*	0.6276(4)	0.8025(9)	0.2325(3)	0.0444(4)	$Ca_{0.77}$+$Sr_{0.23}$

M3	18*b*	0.1443(9)	0.2891(2)	0.3293(3)	0.0394(2)	$Ca_{0.62}$+$Sr_{0.38}$
M4	6*a*	0	0	0.1774(5)	0.0024(6)	$Ca_{0.9}$+$Sr_{0.10}$
M5	6*a*	0	0	-0.0041(6)	0.088(1)	$Cu_{1.0}$
P1	6*a*	0	0	0.2520(9)	0.0064(5)	$P_{1.0}$
P2	18*b*	0.6874(7)	0.839(2)	0.1352(6)	0.0094(6)	$P_{1.0}$
P3	18*b*	0.6488(9)	0.822(4)	0.0329(5)	0.0036(2)	$P_{1.0}$
O1	6*a*	0	0	0.2926(9)	0.0089	$O_{1.0}$
O2	6*a*	0.025(4)	0.154(2)	0.2619(1)	0.0089	$O_{1.0}$
O3	18*b*	0.730(4)	0.912(4)	0.1720(7)	0.0089	$O_{1.0}$
O4	18*b*	0.761(5)	0.784(5)	0.1084(9)	0.0089	$O_{1.0}$
O5	18*b*	0.763(5)	0.746(5)	0.1287(1)	0.0089	$O_{1.0}$
O6	6*a*	0.538(3)	0.833(4)	0.1310(3)	0.0089	$O_{1.0}$
O7	18*b*	0.599(5)	0.922(5)	0.0518(1)	0.0089	$O_{1.0}$
O8	18*b*	0.590(5)	0.672(4)	0.0524(2)	0.0089	$O_{1.0}$
O9	18*b*	0.814(2)	0.924(4)	0.0407(1)	0.0089	$O_{1.0}$
O10	18*b*	0.614(4)	0.828(5)	0.9988(9)	0.0089	$O_{1.0}$

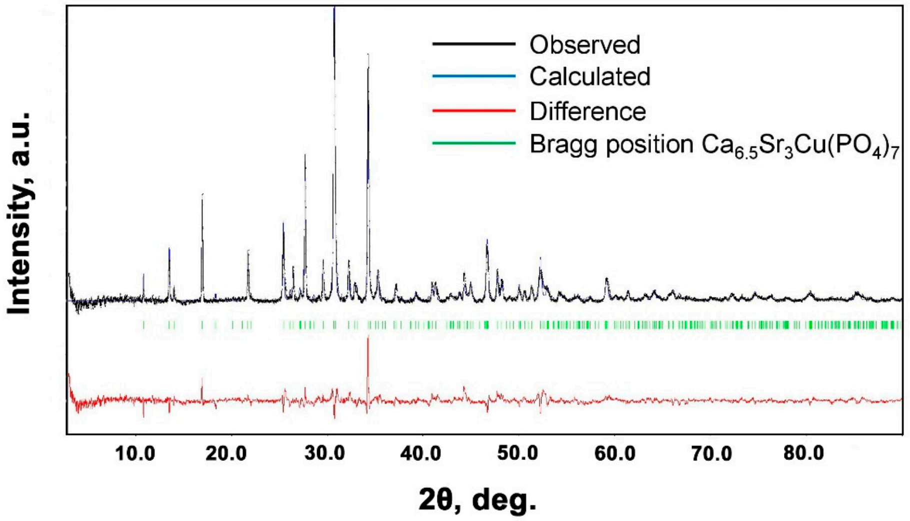

Figure S8. Intensity profiles for the powder X-ray Rietveld refinement of $Ca_{6.5}Sr_3Cu(PO_4)_7$. The observed and calculated profiles are represented in black and blue lines, respectively. The

difference in the profile is plotted at the bottom (red line). Vertical bars indicate the positions of the Bragg reflections (green bars).

Table S10. Atomic coordinates, displacement parameters (Å^2) and site-occupancy factors (SOFs) in the structure of $Ca_6Sr_{3.5}Cu(PO_4)_7$.

Atom	Wyckoff site	*x*	*y*	*z*	U_{iso}, Å^2	SOF
M1	18*b*	0.7244(1)	0.8682(5)	0.4351(2)	0.0148(1)	$Ca_{0.68}$+$Sr_{0.32}$
M2	18*b*	0.6318(1)	0.8137(6)	0.2334(3)	0.0217(8)	$Ca_{0.70}$+$Sr_{0.30}$
M3	18*b*	0.1428(5)	0.2873(8)	0.3310(2)	0.0278(2)	$Ca_{0.54}$+$Sr_{0.46}$
M4	6*a*	0	0	0.1780(7)	0.0169(2)	$Ca_{0.91}$+$Sr_{0.09}$
M5	6*a*	0	0	-0.0010(5)	0.0122(7)	$Cu_{1.0}$
P1	6*a*	0	0	0.2534(9)	0.0436(3)	$P_{1.0}$
P2	18*b*	0.6950(4)	0.8691(6)	0.1298(5)	0.0049(3)	$P_{1.0}$
P3	18*b*	0.6540(9)	0.814(2)	0.0318(5)	0.0012(3)	$P_{1.0}$
O1	6*a*	0	0	0.2938(9)	0.0089	$O_{1.0}$
O2	6*a*	-0.009(3)	0.127(3)	0.2351(8)	0.0089	$O_{1.0}$
O3	18*b*	0.717(3)	0.887(4)	0.1699(5)	0.0089	$O_{1.0}$
O4	18*b*	0.743(5)	0.753(4)	0.1352(9)	0.0089	$O_{1.0}$
O5	18*b*	0.754(5)	0.775(4)	0.1127(9)	0.0089	$O_{1.0}$
O6	6*a*	0.5254(4)	0.786(4)	0.1302(1)	0.0089	$O_{1.0}$
O7	18*b*	0.613(4)	0.923(4)	0.0485(1)	0.0089	$O_{1.0}$
O8	18*b*	0.596(4)	0.668(4)	0.0522(1)	0.0089	$O_{1.0}$
O9	18*b*	0.8117(9)	0.937(3)	0.0410(1)	0.0089	$O_{1.0}$
O10	18*b*	0.606(3)	0.828(6)	0.9937(8)	0.0089	$O_{1.0}$

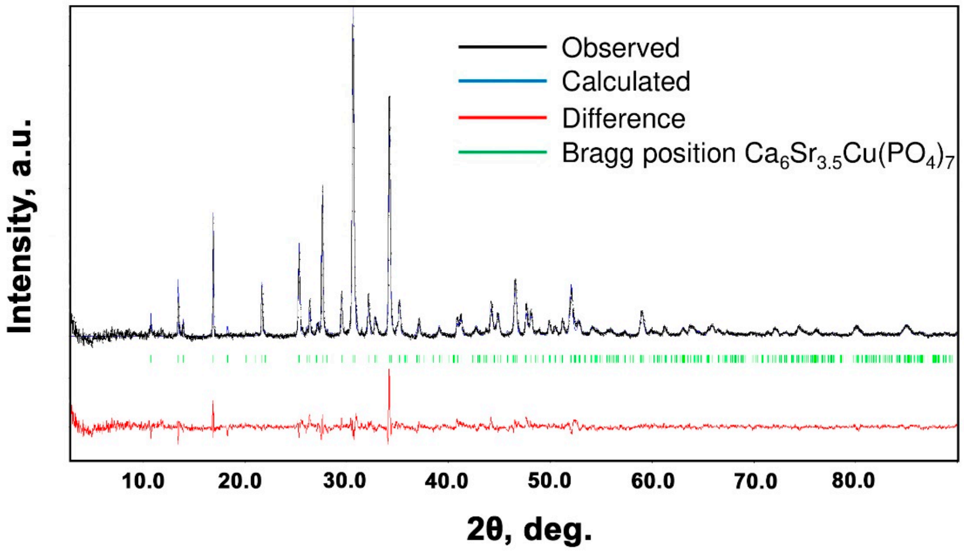

Figure S9. Intensity profiles for the powder X-ray Rietveld refinement of $Ca_6Sr_{3.5}Cu(PO_4)_7$. The observed and calculated profiles are represented in black and blue lines, respectively. The difference in the profile is plotted at the bottom (red line). Vertical bars indicate the positions of the Bragg reflections (green bars).

Table S11. Atomic coordinates, displacement parameters (Å^2) and site-occupancy factors (SOFs) in the structure of $Ca_{5.5}Sr_4Cu(PO_4)_7$.

Atom	Wyckoff site	*x*	*y*	*z*	U_{iso}, Å^2	SOF
M1	18*b*	0.7222(9)	0.8645(6)	0.4355(3)	0.0106(9)	$Ca_{0.65}$+$Sr_{0.35}$
M2	18*b*	0.6336(1)	0.8162(7)	0.2341(3)	0.0099(1)	$Ca_{0.62}$+$Sr_{0.38}$
M3	18*b*	0.1399(4)	0.2875(7)	0.3312(2)	0.0193(7)	$Ca_{0.46}$+$Sr_{0.54}$
M4	6*a*	0	0	0.1815(7)	0.01(2)	$Ca_{0.95}$+$Sr_{0.05}$
M5	6*a*	0	0	0.0004(6)	0.0122(1)	$Cu_{1.0}$
P1	6*a*	0	0	0.2460(3)	0.097(3)	$P_{1.0}$
P2	18*b*	0.7011(9)	0.8711(8)	0.1330(6)	0.042(7)	$P_{1.0}$
P3	18*b*	0.656(2)	0.816(3)	0.0344(6)	0.004(4)	$P_{1.0}$
O1	6*a*	0	0	0.2863(3)	0.0089	$O_{1.0}$
O2	6*a*	-0.022(3)	0.131(2)	0.2373(1)	0.0089	$O_{1.0}$

O3	18*b*	0.709(3)	0.888(4)	0.1733(6)	0.0089	$O_{1.0}$
O4	18*b*	0.759(6)	0.762(5)	0.1310(1)	0.0089	$O_{1.0}$
O5	18*b*	0.738(6)	0.763(5)	0.1140(1)	0.0089	$O_{1.0}$
O6	6*a*	0.5316(9)	0.786(5)	0.1318(1)	0.0089	$O_{1.0}$
O7	18*b*	0.611(4)	0.931(4)	0.0461(2)	0.0089	$O_{1.0}$
O8	18*b*	0.599(5)	0.668(3)	0.0534(2)	0.0089	$O_{1.0}$
O9	18*b*	0.816(2)	0.939(3)	0.0414(1)	0.0089	$O_{1.0}$
O10	18*b*	0.602(3)	0.813(5)	0.9955(1)	0.0089	$O_{1.0}$

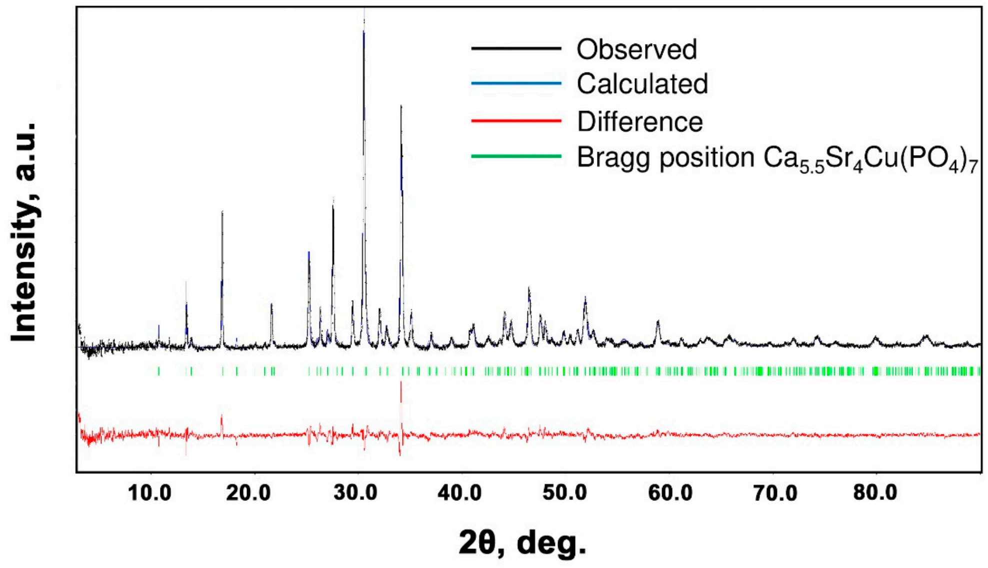

Figure S10. Intensity profiles for the powder X-ray Rietveld refinement of $Ca_{5.5}Sr_4Cu(PO_4)_7$. The observed and calculated profiles are represented in black and blue lines, respectively. The difference in the profile is plotted at the bottom (red line). Vertical bars indicate the positions of the Bragg reflections (green bars).

Table S12. Atomic coordinates, displacement parameters (Å^2) and site-occupancy factors (SOFs) in the structure of $Ca_5Sr_{4.5}Cu(PO_4)_7$.

Atom	Wyckoff site	*x*	*y*	*z*	U_{iso}, Å^2	SOF
M1	18*b*	0.7218(8)	0.8623(6)	0.4361(2)	0.0115(3)	$Ca_{0.55}$+$Sr_{0.45}$
M2	18*b*	0.6321(1)	0.8132(6)	0.2349(2)	0.0115(3)	$Ca_{0.54}$+$Sr_{0.46}$

M3	18*b*	0.1438(5)	0.2928(8)	0.3329(2)	0.0142(8)	$Ca_{0.49}+Sr_{0.51}$
M4	6*a*	0	0	0.1815(7)	0.0052(5)	$Ca_{0.94}+Sr_{0.06}$
M5	6*a*	0	-0.0015(5)	-0.0015(5)	0.0122(5)	$Cu_{1.0}$
P1	6*a*	0	0	0.2482(7)	0.0182(7)	$P_{1.0}$
P2	18*b*	0.6969(6)	0.8660(6)	0.1351(5)	0.0061(1)	$P_{1.0}$
P3	18*b*	0.658(2)	0.817(3)	0.817(3)	0.0084(1)	$P_{1.0}$
O1	6*a*	0	0	0.2883(7)	0.0089	$O_{1.0}$
O2	6*a*	0.049(5)	0.1532(9)	0.2638(8)	0.0089	$O_{1.0}$
O3	18*b*	0.721(3)	0.904(4)	0.1743(5)	0.0089	$O_{1.0}$
O4	18*b*	0.752(7)	0.754(5)	0.1338(5)	0.0089	$O_{1.0}$
O5	18*b*	0.751(7)	0.762(5)	0.1212(5)	0.0089	$O_{1.0}$
O6	6*a*	0.5277(6)	0.786(4)	0.1368(1)	0.0089	$O_{1.0}$
O7	18*b*	0.607(6)	0.925(6)	0.0491(1)	0.0089	$O_{1.0}$
O8	18*b*	0.600(6)	0.673(4)	0.0574(1)	0.0089	$O_{1.0}$
O9	18*b*	0.812(2)	0.932(5)	0.0499(1)	0.0089	$O_{1.0}$
O10	18*b*	0.611(4)	0.823(5)	1.0002(8)	0.0089	$O_{1.0}$

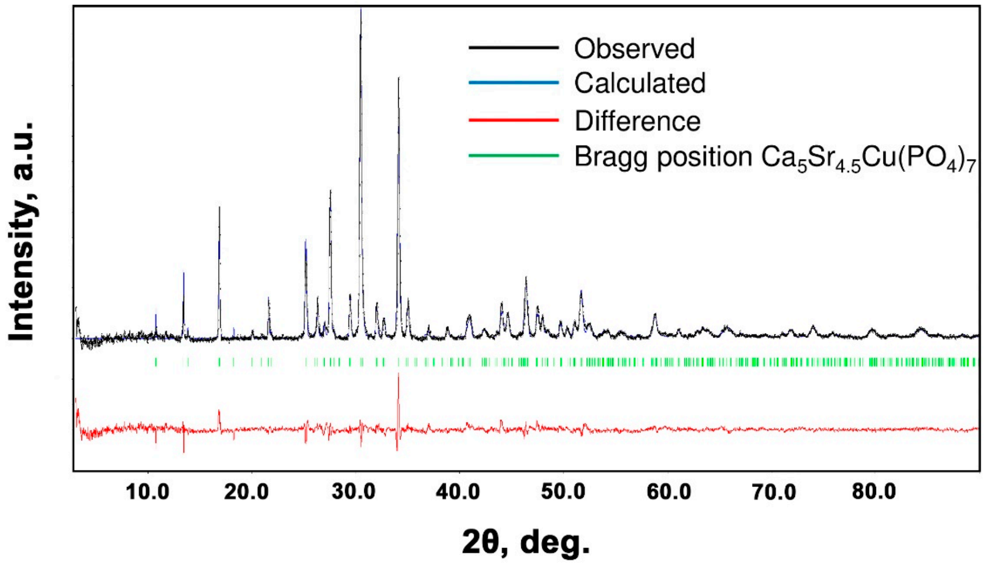

Figure S11. Intensity profiles for the powder X-ray Rietveld refinement of $Ca_5Sr_{4.5}Cu(PO_4)_7$. The observed and calculated profiles are represented in black and blue lines, respectively. The

difference in the profile is plotted at the bottom (red line). Vertical bars indicate the positions of the Bragg reflections (green bars).
